# UK children’s sleep and anxiety during the COVID-19 pandemic

**DOI:** 10.1186/s40359-022-00729-4

**Published:** 2022-03-21

**Authors:** Victoria C. P. Knowland, Elaine van Rijn, M. Gareth Gaskell, Lisa Henderson

**Affiliations:** 1 Department of Psychology, University of York, York, YO10 5DD UK; 2 Present Address: Speech and Language Sciences, Newcastle University, Newcastle upon Tyne, NE1 7RU UK

**Keywords:** Sleep, Anxiety, Childhood, COVID-19

## Abstract

**Background:**

Sleep and mental wellbeing are intimately linked. This relationship is particularly important to understand as it emerges over childhood. Here we take the opportunity that the COVID-19 pandemic, and resulting lockdown in the UK, presented to study sleep-related behaviour and anxiety in school-aged children.

**Methods:**

Parents and children were asked to complete questionnaires towards the start of the UK lockdown in April-to-May of 2020, then again in August of that year (when many restrictions had been lifted). We explored children’s emotional responses to the pandemic and sleep patterns at both time points, from the perspectives of parents and children themselves.

**Results:**

Children’s bedtime anxiety increased at the start of the lockdown as compared to a typical week; however, by August, bedtime anxiety had ameliorated along with children’s COVID-19 related anxiety. Bedtime anxiety predicted how long it took children to fall asleep at night at both the start and the end of the lockdown. Bedtime and wake-up time shifted at the start of lockdown, but interestingly total sleep time was resilient (likely owing to an absence of early school start times) and was not predicted by child anxiety.

**Conclusions:**

These findings further support calls for sleep quality (in particular, time taken to fall asleep) to be taken as a key indicator of mental health in children, particularly under usual circumstances when schools are open and sleep duration may be less resilient.

**Supplementary Information:**

The online version contains supplementary material available at 10.1186/s40359-022-00729-4.

## Introduction

Sleep is closely tied to mental health. Sleep-related problems are experienced by individuals with high levels of anxiety across the lifespan [1, 2], and poor sleep impacts negatively on emotional regulation [3]. Here we aimed to better understand the relationship between anxiety and children’s sleep behaviour by examining how a potentially stressful environmental event (namely, the UK lockdown necessitated by the COVID-19 pandemic in 2020) influenced sleep behaviour and anxiety in children. Utilising parent- and child-reports, we asked if and how anxiety and sleep behaviour changed from the height of lockdown (in the Spring) to later in the year when many of the restrictions on social interaction and daily activities had been lifted. We further asked if any observed changes in these two key health-related variables were related.

### Sleep and anxiety in childhood

A growing literature supports an association between anxiety and sleep-related problems in childhood [4–6]. Across studies of children with clinical anxiety, sleep-related problems are reported in ~ 90% of cases [7–13]. In the community too, correlations are evident, with anxiety being linked to bedtime resistance, fear of sleeping alone, fear of the dark and nightmares in 8–11 year olds [14] and parent-reported anxiety/depression symptoms predicting daytime sleepiness [15].

The relationship between sleep problems and anxiety is likely bi-directional [16]. Not only is anxiety associated with pre-sleep rumination [17], but sleep difficulties can also precede high anxiety. Kelly and El-Sheikh (2014) [18] followed a community sample over five years (from mean age 8.68 years) and found that worse sleep quality and reduced sleep duration predicted greater anxiety over the course of the study, highlighting a role for sleep problems in escalating anxiety symptomology during the transition to adolescence. Sleep problems predict anxiety over time within childhood [19], as well as being a risk factor for anxiety in adulthood [20].

Extant literature suggests that both sleep and anxiety were impacted by COVID-19 in multiple countries and across age groups. In China, insomnia at the peak of the pandemic was seen more severely in women, children, those living in the epicentre and those who were experiencing a high level of threat from the virus, for example those who had or were related to infected individuals [21]. In a sample of 1,630 adults, around a third reported poor sleep during the peak of the first wave in China, with insomnia being significantly predicted by perceived stress [22]; this relationship was mediated by anxiety. In Italy, a sample of 400 adults and young adult students [23] reported later bed times and wake up times, and increased sleep onset latency, as well as a worsening of sleep quality; 34.3% of the sample showed symptoms of anxiety during lockdown. In children and adolescents, national lockdowns have been associated with heightened anxiety [24, 25], either as a result of the fear of illness, illness in others or the effects of confinement and isolation [26]. Sleep behaviours are also known to have altered [27], with 54% of children reported to show sleep disturbance during lockdown. Specific associations between changes in sleep behaviour and increases in anxiety having been demonstrated in Italy [28], Egypt [29] and Spain [30], according to parent report.

Here, we examined whether the first UK national lockdown in 2020 was associated with changes in sleep behaviour in children, and whether any observed changes were related to the experience of heightened anxiety as reported by both parents and children themselves. Establishing a complete picture of the world-wide changes in sleep and mental health faced by young people during the COVID-19 pandemic is essential. This is true both to understand possible long-term sequalae, given that disruption to sleep is a potential route by which anxiety may be exacerbated or prolonged [18], and to be able to put in place better support in the event of future pandemics.

### COVID-19 in the UK

The coronavirus SARS-CoV-2, which causes the acute respiratory disease COVID-19, was first confirmed in the UK on 31st January, 2020. The disease was declared a pandemic by the World Health Organisation on 11th March, and national lockdown came into force in the UK on 26th March, with all schools, leisure establishments and non-essential shops closing, and strict regulations imposed on social interaction and non-essential travel. The first wave of the virus peaked (with respect to reported daily deaths) in mid-April. Data collection for the current study occurred towards the start of the first national lockdown, when virus-related deaths in the first wave were at their highest (967 per day), then in August, by which time restrictions had been reduced and daily death rate had fallen to around 7 per day (according to the UK Government https://www.gov.uk/guidance/coronavirus-covid-19-information-for-the-public). Data were not collected over subsequent waves of the virus.

### Aims of the current study

We considered whether any changes in children’s anxiety levels over the course of the first wave of the COVID-19 pandemic in the UK were associated with any changes observed in sleep behaviour over the same period. This was a valuable opportunity to study sleep and anxiety during a time when there was relatively little daytime structure to constrain sleep patterns for children. Our research questions were as follows: Did children’s sleep behaviour and/or anxiety change at the start of lockdown? Did anxiety and/or sleep change over the course of lockdown (i.e., from April–May to late August)? And finally, did anxiety predict sleep behaviour at the start and/or end of lockdown? To address these questions we invited parents and their children to complete online questionnaires at the start of lockdown (Phase 1) about current (and retrospective) sleep patterns, and anxiety, and again at the end of the summer (Phase 2).

## Method

### Phase 1

**Fig. 1:**
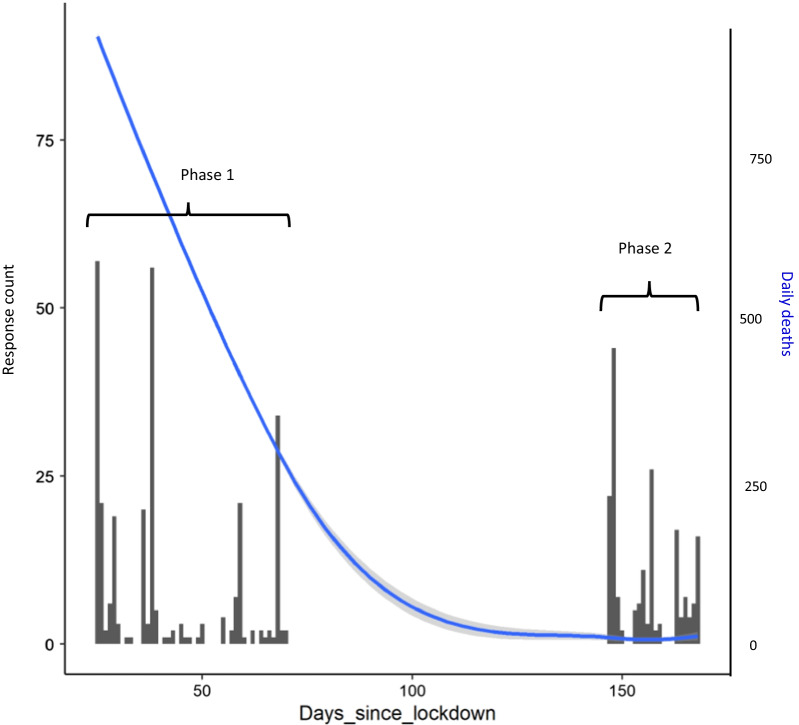
Timing of responses at Phase 1 then Phase 2 relative to the start of lockdown in the UK. The blue line shows 7-day averaged daily death count in the UK, with shaded area showing 95% confidence region. Source: https://coronavirus.data.gov.uk/details/deaths

Questionnaire data were collected via Qualtrics [31]. Participants were recruited through social media, schools and academy trusts, the University of York staff newsletter, and by encouraging participants to share the questionnaire link. Phase 1 went live on 16th April 2020, three and a half weeks after the start of the first UK COVID-19 lockdown, and was closed on 1st June 2020. Phase 2 went live on 17th August 2020 and closed on 6th September 2020, the day before most schools re-opened after the summer (see Fig. 1). The questionnaires took around 10 min to complete. At the end of the Phase 1 questionnaire participants were re-directed to an educational resource about sleep.^1^ Respondents who gave consent were entered into a prize draw to win one of five £60 Amazon vouchers, which were distributed after Phase 2 was completed. At both Phases, the first half of the questionnaire was for the parent/carer and the second was for the child (to be filled in on their own or with a caregiver). Full details of the questionnaires along with responses are provided in Additional file 1. Ethical approval was granted by the Department of Psychology University of York Ethics Committee.

#### Participants

**Fig. 2:**
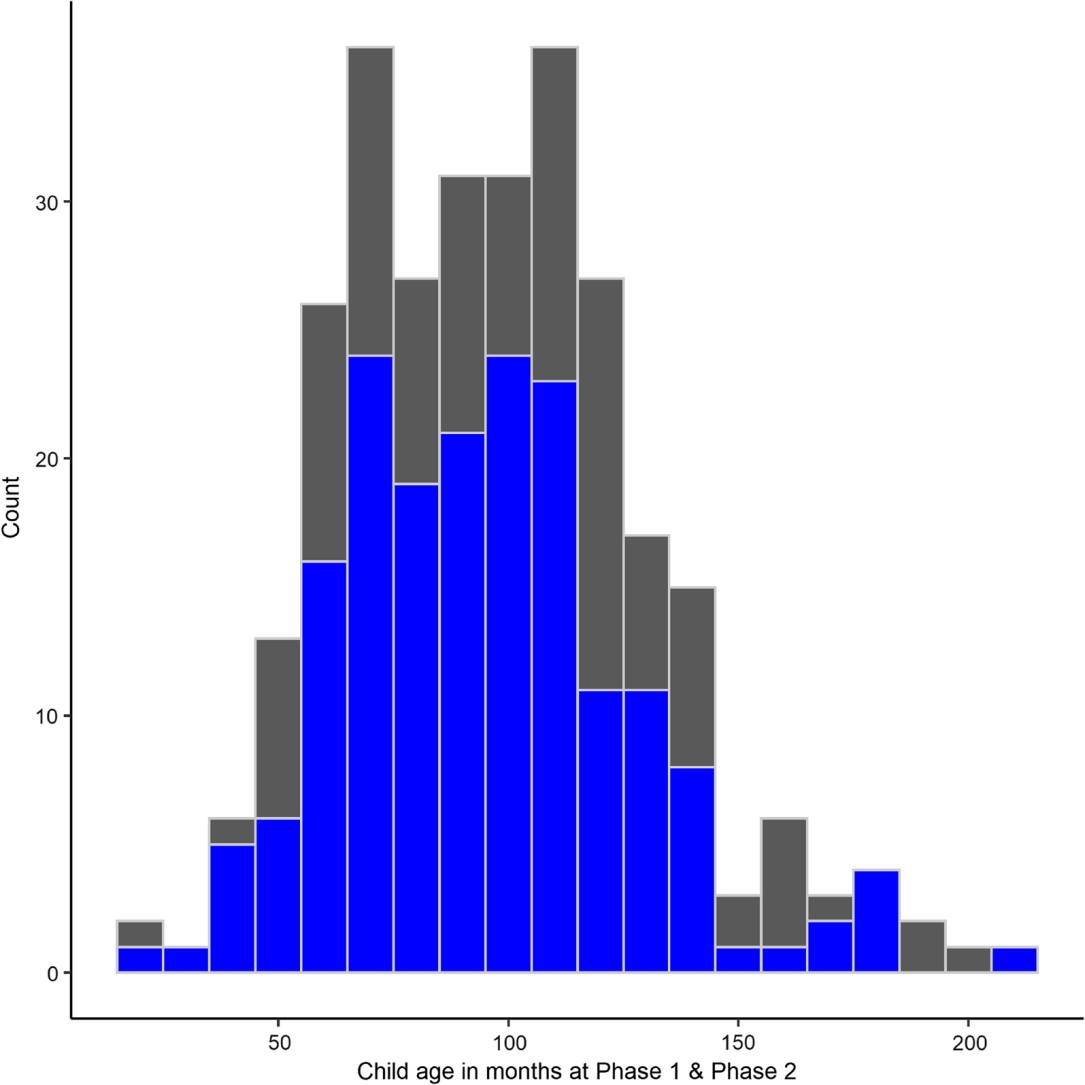
Histogram of the ages of children represented in the survey responses, in months at Phase 1 (grey) & Phase 2 (blue)

At Phase 1, a total of 302 parents/carers (henceforth ‘parents’) responded to at least the parent section of the questionnaire and gave an email address to be re-contacted at Phase 2; of these, 288 reported that they lived in the UK and were consequently including in the analysis. The remaining 14 were excluded from analysis as parents reported that they lived in countries where the number of COVID-19 cases and levels of restriction were different to the UK. The following demographics were reported for children/young people (henceforth ‘children’)^2^ at Phase 1: 52.0% were male and 48.0% female; average age was 97.8 months (8;02 years; *SD* = 34.1 month; see Fig. 2); 8.0% of the sample were reported to have one neurodevelopmental disorder (NDD) either diagnosed or under assessment, and 4.5% had two or more disorders (see Additional file 2: Table A1). Mean national indices of multiple deprivation (IMD) decile for families was 7.1 (*SD* = 2.6). IMD data were moderately negatively skewed (skewness =  − 0.76, kurtosis = 2.63), with more of respondents living in high IMD areas. The mean number of people per bedroom (PPB) was 1.2 (*SD* = 0.4). PPB is a measure of overcrowding [32] and as such taken as an additional measure of economic prosperity. Of the 288 households represented, 15.8% included at least one healthcare worker. Respondents were asked whether anyone in the household had experienced symptoms of COVID-19 (18.5% had) or had tested positive for/received a diagnosis of COVID-19 (1.3% had).

**Table 1:** Parent responses to questions regarding anxiety

	Question	% ‘Not at all anxious’	% ‘A little anxious’	% ‘Somewhat anxious’	% ‘Anxious’	% ‘Very anxious’
Child	Would you typically describe your child as anxious (before Covid-19)?	35.8	38.5	16.3	7.3	2.1
	Did your child get anxious about going to bed before Covid-19?	61.8	27.1	8.0	2.1	1.0
	Does your child get anxious before going to bed at the moment?	51.0 (*59.2*)	22.6 (*21.2*)	9.0 (*11.7*)	11.1 (*5.6*)	6.3 (*2.2*)
	Has your child expressed anxiety about Covid-19?	24.7 (*57.0*)	36.1 (*29.1*)	27.7 (*7.3*)	8.3 (*5.6*)	4.2 (*1.1*)
Parent	Are you typically an anxious person (before Covid-19)?	28.1	35.1	17.7	14.2	4.9
	Do you feel anxious about Covid-19?	6.3 (*27.4*)	32.6 (*44.7*)	35.1 (*17.3*)	16.7 (*6.7*)	9.4 (*3.9*)

Note that exact responses were worded in a way that was appropriate to the question—see the dataset in Additional file 1 for exact wording. Responses at Phase 2 are shown (in italics)

### Phase two

At Phase 2 (August, 2020), all parents who responded to the first questionnaire (N = 302) were invited to fill out the second. A total of 188 did so; of whom 9 lived outside the UK and were therefore excluded from analysis as the countries these respondents reported living in were again experiencing different case numbers and restrictions to the UK. For data analysis at Phase 2, 179 parent participants were therefore included: 48.6% of the children represented were male, 51.4% were female; average age 96.5 months (8;01 years; SD = 29.82 month, see Fig. 2); 11.17% had one or more NDD. The mean number of PPB was 0.92 (*SD* = 0.39; not significantly different to Phase 1, *t* = 1.030). At this second time point, 30.7% reported that someone in their household had experienced symptoms of COVID-19, with 3.4% having a member of the household who had tested positive for/received a diagnosis.

As detailed in Additional file 1, questions were repeated from Phase 1 where appropriate in order to assess changes between timepoints. In addition, parents were asked about changes they had noticed in their children over the course of lockdown.

## Results

### RQ1: Did children’s sleep behaviour and/or anxiety change at the start of lockdown?

#### Parent reported sleep

**Fig. 3:**
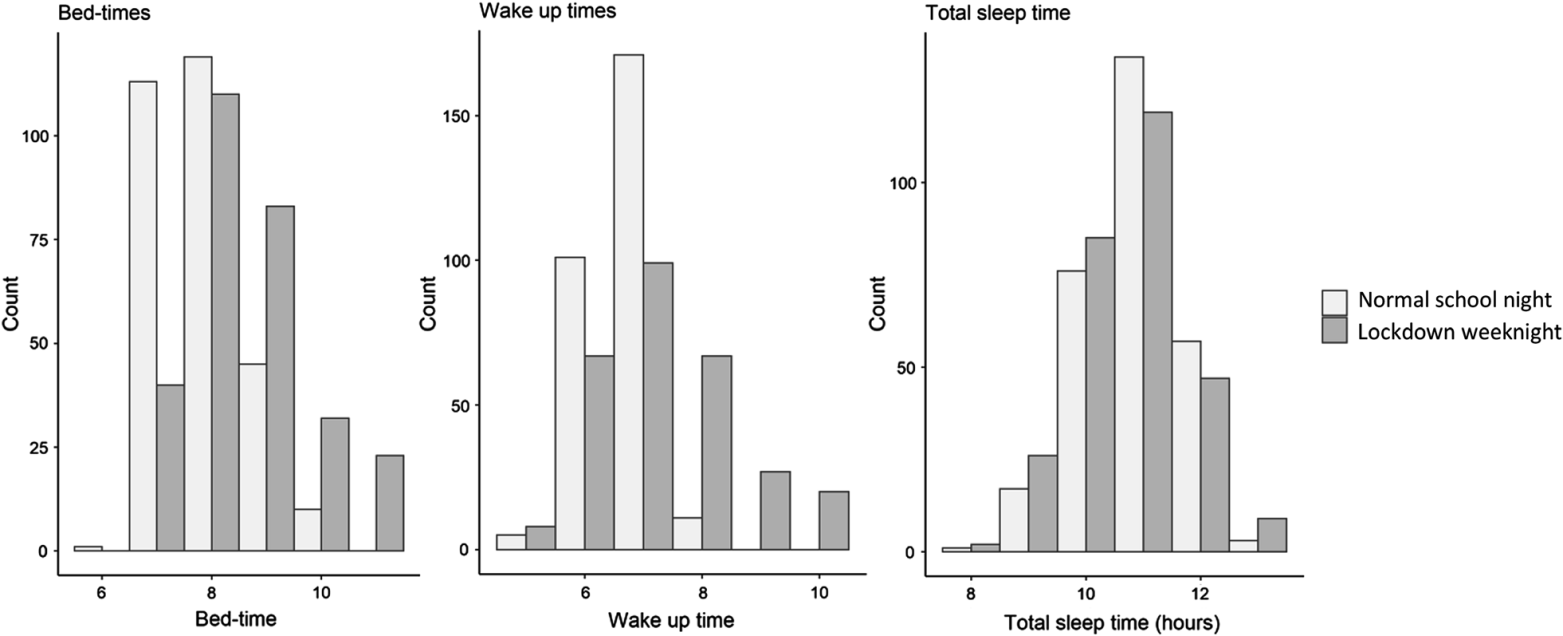
Histograms showing bedtimes, wake-up times and total sleep times of children during a normal school night and during a lockdown weeknight

Parents were asked questions about their child’s sleep ‘during the past week (while schools were closed)’ and also ‘during a typical week (when schools were open)’ before the COVID-19 pandemic. Parents were asked *What time does your child go to bed on weeknights?* and *What time does your child wake up on weekdays?*. Options were given to the nearest hour as bedtimes are known to vary substantially [33] and parents are approximate in their estimates of their children’s sleep times even over the primary school years [34]. Mean bed time became significantly later at the start of lockdown (mean = 8:37 pm, *SD* = 67 min) relative to a typical week of school (mean = 7:50 pm, *SD* = 49 min; *t*(529.8) =  − 9.675, *p* < 0.001) as did mean wake-up time (6:39am ( *SD* = 35 min) to 7:21am (*SD* = 50 min); *t*(409.83) =  − 8.755, *p* < 0.001) (Fig. 3). However, total sleep time (TST), calculated to the nearest hour based on bed time and wake-up times, did not change: TST during a typical week was 10 h 50 min (*SD* = 52 min), and during the surveyed lockdown week, 10 h 43 min (*SD* = 61 min). Age in months predicted change in both bed time (B =  − 0.003, *t* =  − 2.13, *p* = 0.034) and wake-up time (B =  − 0.012, *t* =  − 6.57, *p* < 0.001), with younger children being less likely to show change in patterns of behaviour.

#### Parent reported anxiety

Parent-reported anxiety was recorded for both parents themselves and their children (see Table 1). A notable shift was evident in reported bedtime anxiety, with 61.8% of children being described as ‘not at all anxious’ at bedtime before the lockdown, dropping to 51.0% at the start of lockdown.

We considered whether the change in bedtime anxiety from before lockdown to the start of lockdown could be predicted by the child’s general anxiety level, the extent to which they had expressed worry about COVID-19 to their parents, days since lockdown began, the child’s age and whether anyone in the household had experienced symptoms at the time of parents’ responses. In an ordinal logistic regression, every step up in children’s expressed COVID-19 anxiety increased the proportional odds of them showing an increase in bedtime anxiety. Most notably, with a shift in a child’s COVID-19 related anxiety from ‘Quite a bit’ to ‘A lot’, children were 56.3X more likely to show an increased level of bedtime anxiety (Table 2).

**Table 2:** Proportional odds ratios with 97.5% CIs for changes in bedtime anxiety predicted by change in general (parent reported) child anxiety, expressed anxiety about covid, time since lockdown in days, child age in months and whether someone in the household has symptoms of the virus (yes or no)

	Term	OR	Lower 97.5% CI	Upper 97.5% CI	*p*
Would you typically describe your child as anxious (before Covid-19)?	Not at all anxious-> A little anxious	0.94	0.52	1.71	0.846
	A little anxious-> Somewhat anxious	0.80	0.36	1.76	0.575
	Somewhat anxious-> Anxious	1.15	0.41	3.14	0.794
	Anxious-> Very anxious	0.05	0.00	0.54	0.017*
Has your child expressed anxiety about COVID-19?	No anxiety-> A little anxiety	1.53	0.74	3.23	0.258
	A little anxiety-> Some anxiety	5.93	2.76	13.24	< 0.001***
	Some anxiety-> Quite a bit of anxiety	10.60	3.69	30.87	< 0.001***
	Quite a bit of anxiety-> A lot of anxiety	56.30	14.59	233.99	< 0.001***
	Days since lockdown	1.02	1.00	1.03	0.078
	Age in months	1.00	0.99	1.01	0.588
	Household symptoms	0.70	0.35	1.36	0.300

*Significant at *p* < 0.05, ***Significant at *p* < 0.001

### RQ2: Did anxiety and/or sleep change over the course of lockdown?

#### Sleep

At Phase 2, mean bed time was 8:32 pm (*SD* = 64 min), and wake up time was 7:15am (*SD* = 66 min), with a mean TST of 10 h 43 min (*SD* = 60 min). Compared with the start of lockdown none of these values significantly changed (bed time *t*(355.62) = 0.957, *p* = 0.339; wake up time *t*(353.9) = 0, *p* = 1.000; TST *t*(355.79) = -1.016, *p* = 0.310).

**Fig. 4:**
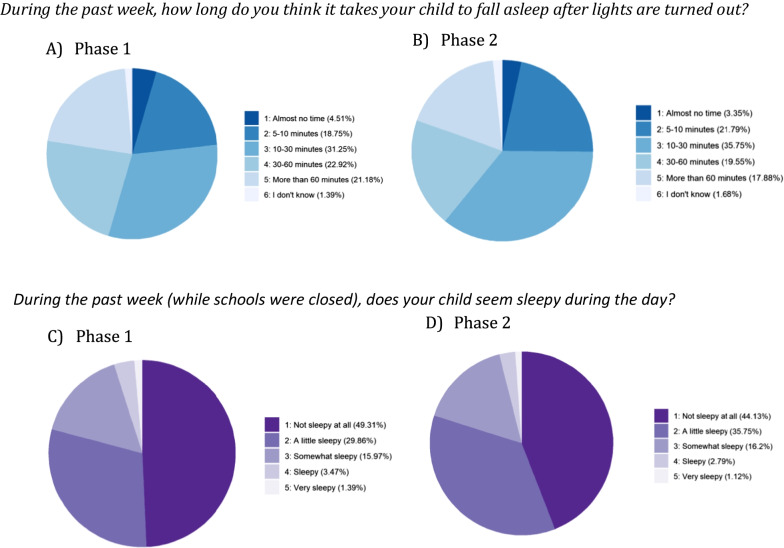
Parent-reported sleep onset latency at **A** Phase 1 (n = 288) and **B** Phase 2 (n = 179). Parent-reported daytime Sleepiness at **C** Phase 1 (n = 288) and **D** Phase 2 (n = 179)

At both phases, parents were asked additional questions regarding their child’s sleep: *How long do you think it takes your child to fall asleep after lights are turned out?* (this question will hereafter be referred to as sleep onset latency; SOL); and *Does your child seem sleepy during the day?* (for responses see Fig. 4). Finally, relating to sleep behaviour, parents were asked *Are you currently worried about your child's sleep?:* at Phase 1, 39.2% reported ‘yes’, with the remaining 60.8% reporting ‘no’. At Phase 2, 38.0% reported that ‘yes’ they were worried, with the remaining 62.0% reporting ‘no’.

No significant differences were evident from the start to the end of lockdown for daytime sleepiness (for *n* = 179; *V* = 1750.5, *p* = 0.973) or whether parents were worried about their child’s sleep (*V* = 644, *p* = 0.227) but a difference did emerge for SOL, for *n* = 176, *V* = 1418, *p* = 0.024, with median response at both phases ‘10–30 min’ (option 3) but with a larger IQR at Phase 2 (*IQR* = 1.0 vs *IQR* = 1.5); as is evident in Fig. 4, fewer parents reported that their children took ‘30–60 min’ or ‘more than 60 min’ to fall asleep at Phase 2 compared to Phase 1.

Parents were asked if they felt their child’s sleep had changed during lockdown, 43.0% of respondents said ‘yes’, 25.7% ‘maybe’ and 31.3% ‘no’. Those parents who reported that ‘yes’ or ‘maybe’ their child’s sleep had changed (68.7% of the whole sample), were asked to select from one or more of seven possible reasons for that change: ‘Not tired in the evenings’ (35.0%); ‘Taking longer to fall asleep’ (66.7%); ‘Needing someone else in the room’ (30.1%); ‘Waking in the night’ (34.1%); ‘Nightmares/night terrors’ (22.0%); ‘Early morning waking’ (17.9%); ‘Daytime sleepiness’(18.7%); ‘Other’ (15.4%). Parents who said their child’s sleep changed over lockdown were then asked if their child’s sleep had returned to normal. Only 7.3% said ‘yes’, with 39.8% reporting ‘somewhat’ and 52.8% ‘no’.

Children themselves were asked if they would be happy to complete a few questions regarding their sleep and feelings about the pandemic; 13.2% did not consent to answer the questions and were consequently not asked to complete any. Where a percentage of ‘no response’ is given, this does not include those who opted to not respond to any questions in this section; rather, responses are detailed here for the 243 children who answered at least some questions. Children were asked questions about their sleep at both phases (Figure A1 in Additional file 2): *At the moment, do you feel you get enough sleep?* and *At the moment, how sleepy do you feel during the day?*; and *After your lights are turned off, do you spend a long time thinking or worrying about things?* Children’s responses to whether they get enough sleep did not significantly change from Phase 1 to Phase 2, based on 137 respondents (*V* = 1509.5, *p* = 0.968). For child-reported SOL, 134 respondents answered at both phases; at both points the median response was ‘I spend some time thinking’, but an increase in variability resulted in a significant change between phases (Phase 1 *IQR* = 1.0; Phase 2 *IQR* = 1.5, *V* = 1226.5, *p* = 0.004). At Phase 2, fewer children reported spending ‘ages’ thinking (15.3% to 8.2%) and more report going ‘straight to sleep’ (6.4% to 11.4%). The correlation between child-reported enough sleep and daytime sleepiness was strong and significant at both phases (at Phase 1:, *r*_*s*_ = 0.53, *p* < 0.001, and at Phase 2: *r*_*s*_ = 0.71, *p* < 0.001), with those who reported not getting enough sleep also reporting higher levels of daytime sleepiness.

Children were asked *How many times do you think you wake up in the night?* Mean response at Phase 2 (134 responses) was 1.37 (*SD* = 1.27), significantly less than at Phase 1, where mean = 1.63 (*SD* = 1.48), *t*(133) =  − 2.49, *p* = 0.014. Finally regarding wake after sleep onset, children were asked *If you wake in the night does it take you a long time to get back to sleep?*: again, this measure changed significantly from Phase 1 to Phase 2 for the 134 respondents who answered this question twice. At Phase 1 median response = ‘some time’ (*IQR* = 3.0) to Phase 2 median response = ‘very little time’ (*IQR* = 2.0), *V* = 1133.5, *p* = 0.003.

#### Anxiety

At Phase 2, parents were asked about current bedtime anxiety and COVID-related anxiety in their child, as well as their own current COVID-19-related anxiety (Table 1). All three of these anxiety measures improved significantly from Phase 1 to Phase 2. Child bedtime anxiety improved from a median response ‘a little anxious’ (*IQR* = 2) to a median response ‘not at all anxious’ (*IQR* = 1.0; *n* = 179); *V* = 821, *p* < 0.001. Children’s anxiety about COVID-19 changed from a median response ‘a little anxiety’ (*IQR* = 1) to ‘no anxiety’ (*IQR* = 1.0), for 179 respondents, *V* = 604, *p* < 0.001. Finally, parental anxiety about COVID-19 also decreased, from a median response ‘somewhat anxious’ (*IQR* = 2.0) to a median response ‘a little anxious’ (*IQR* = 2.0), for 179 respondents, *V* = 834, *p* < 0.001.

Children were asked about anxiety: *During the past week, did you feel scared or worried for no particular reason?* and *During the past week, did you worry about the virus?*. Responses regarding general worry did not significantly change from Phase 1 to Phase 2 (*V* = 1419, *p* = 0.067), while COVID-19-specific worry did, with median response at Phase 1 being I ‘sometimes’ worry, to I ‘rarely’ worry at Phase 2 (Phase 1 *IQR* = 2.0, Phase 2 *IQR* = 2.0): *V* = 966, *p* < 0.001 (see Additional file 2: Table A2 for full breakdown of responses).

### RQ3: Did anxiety predict sleep behaviour at the start and/or end of lockdown?

#### Phase 1

**Fig. 5:**
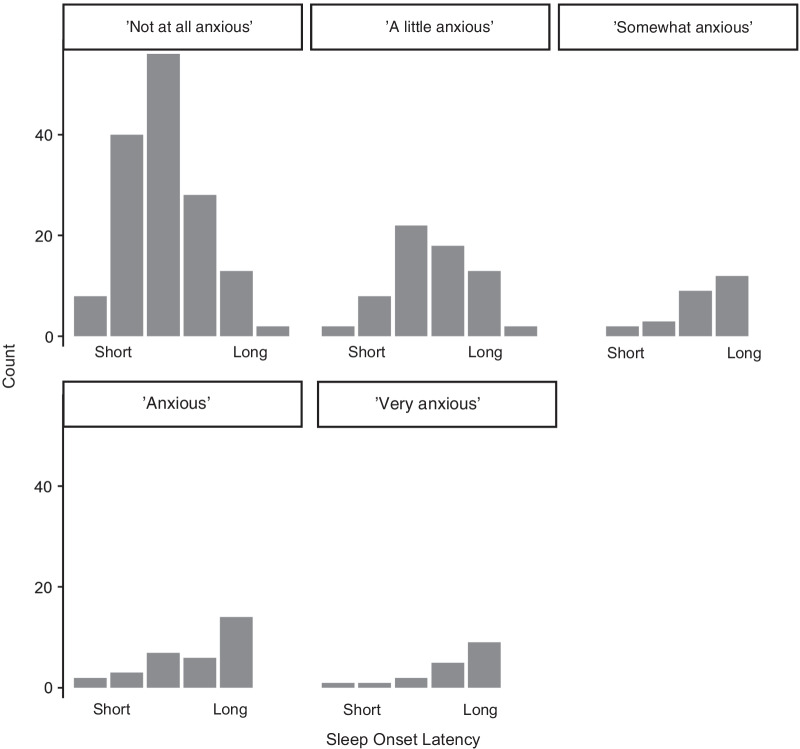
Frequency count for each response option of sleep onset latency from Short to Long (representing ‘Almost no time’, ‘5–10 min’, ‘10–30 min’,’30–60 min’, to ‘More than 60 min’), as a function of each response option for bedtime anxiety (from ‘Not at all anxious’ to ‘Very anxious’)

We asked whether children’s TST or SOL, as reported by their parents, could be predicted by children’s anxiety levels at bedtime, along with household symptoms, age, PPB and whether the child had been diagnosed with or been referred for an NDD. Table 3 gives the results of ordinal logistic regressions, suggesting that bedtime anxiety is the best predictor of SOL during lockdown, with every step up in children’s bedtime anxiety increasing the proportional odds of it taking longer for them to fall asleep after lights out (Fig. 5). Conversely, age was the most reliable predictor of TST.

**Table 3:** Ordinal logistic regression model predicting parent-reported Total Sleep Time (TST) and Sleep Onset Latency (SOL) at Phase 1 for 288 children

		TST Phase 1				SOL Phase 1			
		OR	2.5	97.5	*p*	OR	2.5	97.5	*p*
Does your child get anxious before going to bed at the moment?	Not at all - A little	0.63	0.36	1.11	0.107	2.39	1.39	4.14	0.002**
	A little - Somewhat	0.97	0.41	2.28	0.944	6.24	2.79	14.25	< 0.001***
	Somewhat - Anxious	0.58	0.27	1.27	0.174	3.49	1.53	8.01	0.003**
	Anxious - Very	1.25	0.46	3.43	0.656	4.93	1.73	14.21	0.003**
Has your child expressed anxiety about Covid-19?	No - A little	0.89	0.50	1.57	0.689	1.39	0.79	2.46	0.254
	A little - Some	1.08	0.57	2.04	0.810	1.30	0.69	2.45	0.420
	Some - Quite a bit	1.76	0.67	4.61	0.248	2.46	0.94	6.48	0.067
	Quite a bit - A lot	0.26	0.07	0.93	0.037*	2.29	0.63	8.41	0.208
	Household symptoms	1.48	0.85	2.58	0.169	0.98	0.56	1.71	0.931
	Days since lockdown	1.00	0.98	1.01	0.562	1.01	0.99	1.02	0.472
	Age in months	0.99	0.98	1.00	0.010*	1.01	1.00	1.01	0.073
	PPB	0.70	0.34	1.44	0.329	0.78	0.43	1.42	0.413
	NDD	0.55	0.28	1.08	0.083	1.05	0.53	2.09	0.884

**Significant at *p* < 0.01, ***Significant at *p* < 0.001

As the relationships between parent-reported child anxiety and sleep were strong, we assessed the relationships between worry and sleep parameters as reported by children themselves at Phase 1 (Table 4). The correlations between worry and child-reported sleep onset latency were particularly strong, similar to the parent-reported data.

**Table 4:** Spearman’s Rho correlations between questions regarding sleep behaviour and worry answered by children at Phase 1

	Sleep onset latency	Daytime sleepiness	Nightwakings	SOL after nightwaking
*During the past week, did you feel scared or worried for no particular reason?*	r_s_ = .37 *p* < 0.001	r_s_ = .30 *p* < 0.001	r_s_ = .35 *p* < 0.001	r_s_ = .32 *p* < 0.001
*During the past week, did you worry about the virus?*	r_s_ = .33 *p* < 0.001	r_s_ = .18 *p* = 0.007	r_s_ = .26 *p* < 0.001	r_s_ = .24 *p* < 0.001

n = 243

#### Phase 2

To establish whether anxiety at Phase 2 continued to predict children’s sleep parameters, a second set of regression models were as run predicting parent-reported SOL and TST at Phase 2, with child bedtime anxiety, and child anxiety about COVID-19 at Phase 2, along with whether the household reported symptoms at Phase 2, whether the child had a NDD, PPB and child age at Phase 2 as predictors (see Table 5). Bedtime anxiety remained the only predictor of SOL at Phase 2, as it was at Phase 1, although odds ratios were lower. For TST, age remained a significant predictor, along with a shift in bedtime anxiety from ‘somewhat anxious’ to ‘anxious’, which was associated with shorter TST.

**Table 5:** Ordinal logistic regression model predicting parent-reported Total Sleep Time (TST) and Sleep Onset Latency (SOL) at Phase 2 for 179 children

		TST Phase 2				SOL_Phase 2			
		OR	Lower 97.5%CI	Upper 97.5%CI	*p*	OR	Lower 97.5%CI	Upper 97.5%CI	*p*
Does your child get anxious before going to bed at the moment?	Not at all - A little	0.84	0.40	1.75	0.649	2.27	1.12	4.62	0.023*
	A little - Somewhat	0.69	0.23	2.01	0.499	4.26	1.61	11.62	0.004**
	Somewhat - Anxious	0.08	0.01	0.37	0.002**	4.57	1.10	19.27	0.035*
	Anxious - Very	0.35	0.04	2.42	0.297	2.30	0.35	15.70	0.381
Has your child expressed anxiety about Covid-19?	No - A little	0.64	0.33	1.24	0.188	1.33	0.71	2.50	0.381
	A little - Some	2.08	0.54	7.83	0.282	1.28	0.40	4.04	0.669
	Some - Quite a bit	1.12	0.25	4.92	0.878	1.86	0.44	7.70	0.389
	Quite a bit - A lot	8.58	0.52	244.37	0.138	0.49	0.02	11.40	0.632
	Household symptoms	0.97	0.52	1.79	0.918	0.92	0.50	1.67	0.776
	Age in months	0.99	0.98	1.00	0.003**	1.00	0.99	1.01	0.796
	PPB	0.77	0.29	2.04	0.598	1.14	0.44	2.97	0.780
	NDD	2.69	0.99	7.22	0.050*	2.26	0.84	6.07	0.102

*Significant at *p* < 0.05, **Significant at *p* < 0.01

## Discussion

Given the established links between anxiety and sleep behaviour in children, the aim of the current paper was to present a broad picture of sleep and anxiety in UK children over the course of the first COVID-19 lockdown. The specific research questions addressed were: Did children’s sleep behaviour and/or anxiety change at the start of lockdown? Did anxiety and/or sleep change over the course of the first lockdown? And finally, did anxiety predict sleep behaviour at the start and/or end of lockdown? To address these questions, parents and their children were invited to complete online questionnaires at the start of the first UK lockdown and again at the end, when case numbers had fallen and restrictions on social interaction were beginning to lift.

### Findings

In the present sample of UK children, parents reported that sleep and anxiety changed at the start of lockdown compared to a typical school week. Mean reported bed time became significantly later during the surveyed week at the start of lockdown relative to a typical week of school (by a mean of 47 min) as did mean wake up time (by a mean of 42 min), though total sleep time did not change. We also saw a notable shift in bedtime anxiety, with 10.8% fewer children being rated as ‘not at all’ anxious at bedtime at the start of lockdown compared to a typical week. This change in bedtime anxiety was strongly predicted by anxiety expressed about COVID-19, but not by parental-rated general anxiety.

According to parent-report, children’s bedtime anxiety improved over the course of lockdown. COVID-19-related anxiety also improved in children according to both parent and self-report. However, no significant shifts were seen from the start of lockdown to the end in bed time, wake-up time, total sleep time, daytime sleepiness or whether parents are worried about their child’s sleep. The only sleep parameters that changed over this period were parent-reported and self-reported sleep onset latency, with fewer parents reporting that their children took’30–60 min’ or ‘more than 60 min’ to fall asleep at Phase 2 compared to Phase 1, and fewer children reporting that they ‘spend ages thinking’ before falling alseep. Children also reported fewer night time awakenings and taking less time to get back to sleep after waking in the night. At Phase 2, 68.7% of parents reported that their child’s sleep had or maybe had changed during lockdown, with the most popular responses as to why being ‘taking longer to fall asleep’, ‘not tired in the evening’, ‘waking in the night’ and ‘needing someone in the room to fall asleep’. When asked if their child’s sleep had returned to normal, only 7.3% said ‘yes’.

Bedtime anxiety predicted sleep onset latency at both Phase 1 and Phase 2. Interestingly however, bedtime anxiety did not predict total sleep time, suggesting that with no school to attend, children were able to make up for later nights by sleeping for longer in the morning.

### Anxiety and sleep behaviour in children

Our data suggest that anxiety relating to COVID-19 was associated with UK children’s sleep-related behaviour. Sleep onset latency, which was predicted by bedtime anxiety, improved over lockdown, though parents still perceived sleep-related problems, possibly reflecting parameters more associated with change in routine (e.g., the time at which children went to bed) or possibly to other signs of lingering anxiety (e.g., needing someone to stay in the room while falling asleep).

It is notable that total sleep time was not influenced by bedtime anxiety in these data and was not observed to change over lockdown owing to children waking up later in the morning. A lack of change in total sleep time through COVID-19 lockdown has been demonstrated elsewhere with an adolescent sample. For example, Gruber et al. (2020) [35] conducted semi-structured phone interviews with adolescents in Canada and showed a two-hour shift in sleep patterns but with no disruption to sleep duration, indeed they demonstrated longer sleep duration and better sleep quality. Delayed bedtime and wake times, with no reduction in total sleep time, have also been demonstrated in pre-schoolers in China [36] and school age children in Italy [28]. This resilience of total sleep time is encouraging in the light of evidence to suggest that sleep plays a protective role against the impact of negative emotional experiences [3, 37].

If heightened anxiety is experienced during the normal school term, time asleep could be diminished by increased sleep onset latency. So while extant evidence suggests that the causal links between sleep and anxiety are bi-directional, our data suggest that this may only be true in circumstances where there is no potential for the adjustment of daytime schedules. Furthermore, under circumstances where children and young people were able to adjust their daytime schedules, those who experience anxiety should see the biggest gains with respect to sleep duration.

The current data suggest that older children and adolescents were more likely to show a delay in sleep patterns over the course of lockdown; a finding which is mirrored elsewhere [30]. This age bracket has also been particularly prone to increased levels of anxiety during the pandemic [25, 30]. It is therefore important that mental health professionals consider the role that sleep might be playing in perpetuating any persistent pandemic-related anxiety [18] seen in this population. Encouragingly, social support was highlighted as a protective factor against insomnia for adolescents and young adults in China during the pandemic [38].

### Limitations

This study set out to capture a broad picture of correlations between sleep and anxiety in children in the UK over the COVID-19 lockdown, and as such we acknowledge that the measures of sleep and anxiety were approximate and subjective. Accurate subjective, parent-reported measures of sleep, especially sleep after lights out, are difficult to obtain with a broad age range of children and young people. We therefore opted to leave the wording of the questionnaire vague (e.g., *what time does your child go to bed?*) and consider responses as approximations. While it is possible that that objective measures would have resulted in even stronger correlational relationships, subjective measurements can sometimes be more revealing than objective measures. For example, self-reported sleepiness has been shown to be a stronger predictor of school performance than sleep duration in children and adolescents [39]. Other difficulties include the relatively high SES of the sample, and its self-selecting nature, especially at Phase 2.

While we were not able to consider the internal reliability of our questionnaire (in the interests of brevity), we do see indications of reliability with strong correlations between responses at Phase 1 and Phase 2 (see Additional file 2: Table A3), and strong correlations between self-reported daytime sleepiness and whether children felt they were getting enough sleep at both Phase 1 and Phase 2.

Finally, numerous other variables may well have influenced both sleep and anxiety over the manipulation period, including seasonal effects and economic factors, especially the latter given that a recession was declared in the UK on 12th August, shortly before phase two of the study went live (ONS, 2020); family stress may have been easing with respect to infection rate at the same time as increasing as a result of financial pressure and looming economic crisis.

### Conclusions

The COVID-19 pandemic provided an opportunity to study the correlative relationship between children’s anxiety and sleep behaviour. Here we have seen that bedtime anxiety initially increased at the start of lockdown, then decreased along with COVID-19-related anxiety, and that bedtime anxiety predicted how long it took children to fall asleep at night. Total sleep time was notably resilient in the face of heightened anxiety in children. Change in certain aspects of sleep behaviour as estimated by both parents and self-report may be a useful marker of the stress status of children in response to health crisis. Our data suggest that sleep onset latency might be a useful marker, while total sleep time would not. A key message from the Sleep Council and the Sleep Charity’s Sleep Manifesto (www.sleepcouncil.org.uk) is that sleep quality should be taken as a key indicator of health and wellbeing; checking in with children as to how they are sleeping could provide a window into their mental health.

## Supplementary Information

**Additional file 1**: This file contains the questions asked in the Phase 1 and Phase 2 questionnaires, notes on variable coding and a full data set.

**Additional file 2**: This file contains supplementary materials as listed in the manuscript.

## Data Availability

The dataset supporting the conclusions of this article is included within the article and its Additional files.

## Footnotes

1 30.2% of those dyads who responded to Phase2 completed the educational sleep resource and spent more than 60 s looking at it. To check that this did not have an influence on change in behavior over lockdown we considered group differences in time spent with screens before bed and time spent outside (the two aspects of sleep hygiene that were discussed in the resource), as well as bed time. There were no differences in changes on these measures between those who did and did not complete the resource (change in time with screens, t = -0.73, *p* > .4; change in time outside, t = 0.47, *p* > .6; change in bed time, t = -1.08, *p* > .2). There is therefore no reason to suspect that the sleep resource had an impact on the variables measured at Phase 2.

2 Although some of the young people who responded to the questionnaire were adolescents, we use the term ‘children’ here throughout for simplicity.
