# Supplementary material for: UK children’s sleep and anxiety during the COVID-19 pandemic
Source: BMC Psychol. 2022 Mar 21;10:76. doi: 10.1186/s40359-022-00729-4 (PMC8936042; doi:10.1186/s40359-022-00729-4)
Supplement: Supplementary file 2 — Additional file 2: This file contains supplementary materials as listed in the manuscript. [file 40359_2022_729_MOESM2_ESM.docx]

**Additional file 2: Supplementary materials**

|  | Referred for (%) | Diagnosed with (%) | Expected prevalence(%) |
| --- | --- | --- | --- |
| ADHD | 2.7 | 1.4 | 2.2 |
| Dyslexia | 3.1 | 2.4 | 3-10 |
| Language disorder | 1.0 | 1.0 | 7.6 |
| ASD | 5.6 | 1.7 | 1.6 |
| Other NDD | 1.0 | 3.1 | -- |

**Table A1.** Percentage of parents who reported their child had been referred for or had been diagnosed with a neurodevelopmental disorder (NDD). Expected prevalence rates for the UK are also given, based on best available literature: ADHD^1^; ASD^2^; language disorder^3^; Dyslexia^4^.

*At the moment, do you feel you get enough sleep?*

1. Phase 1


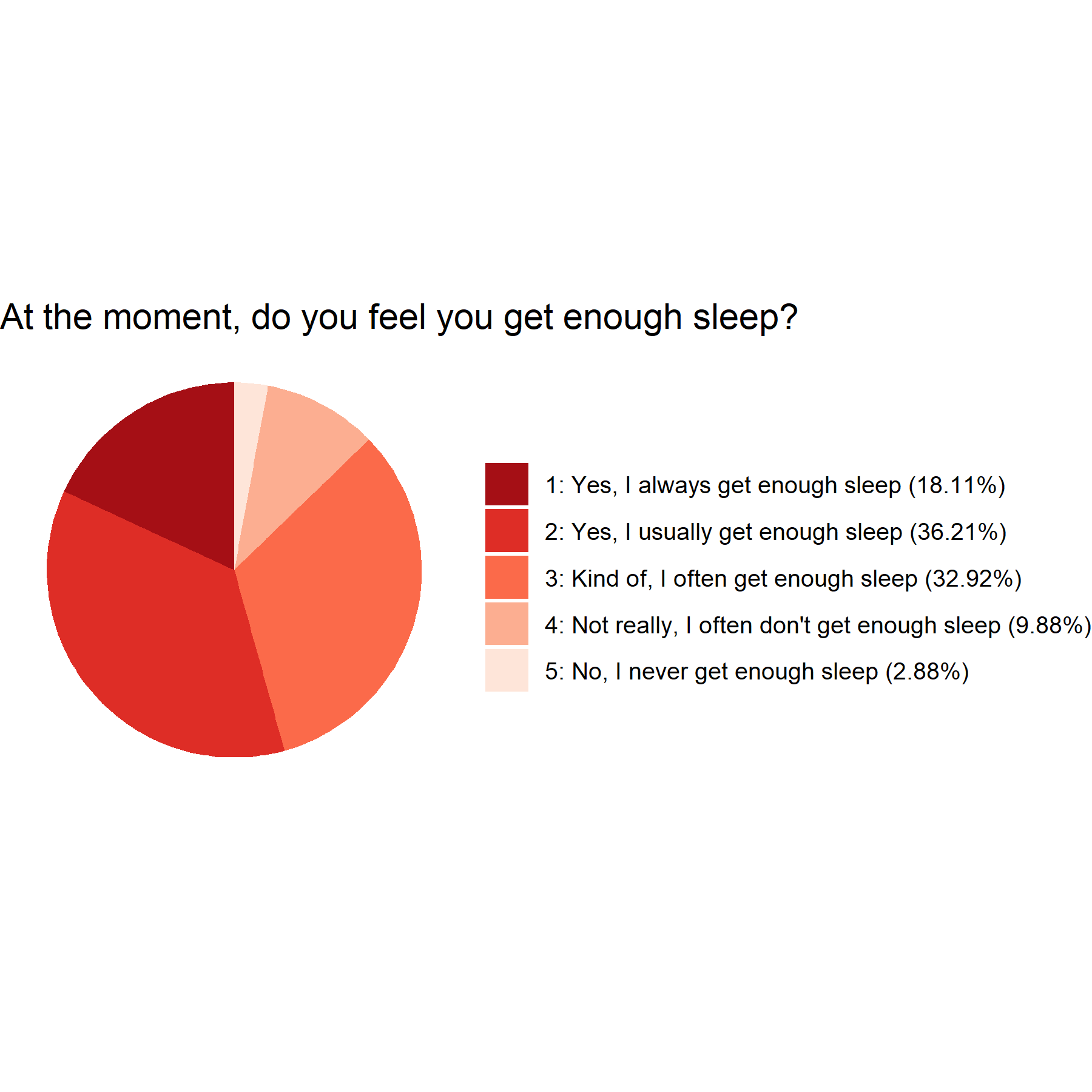

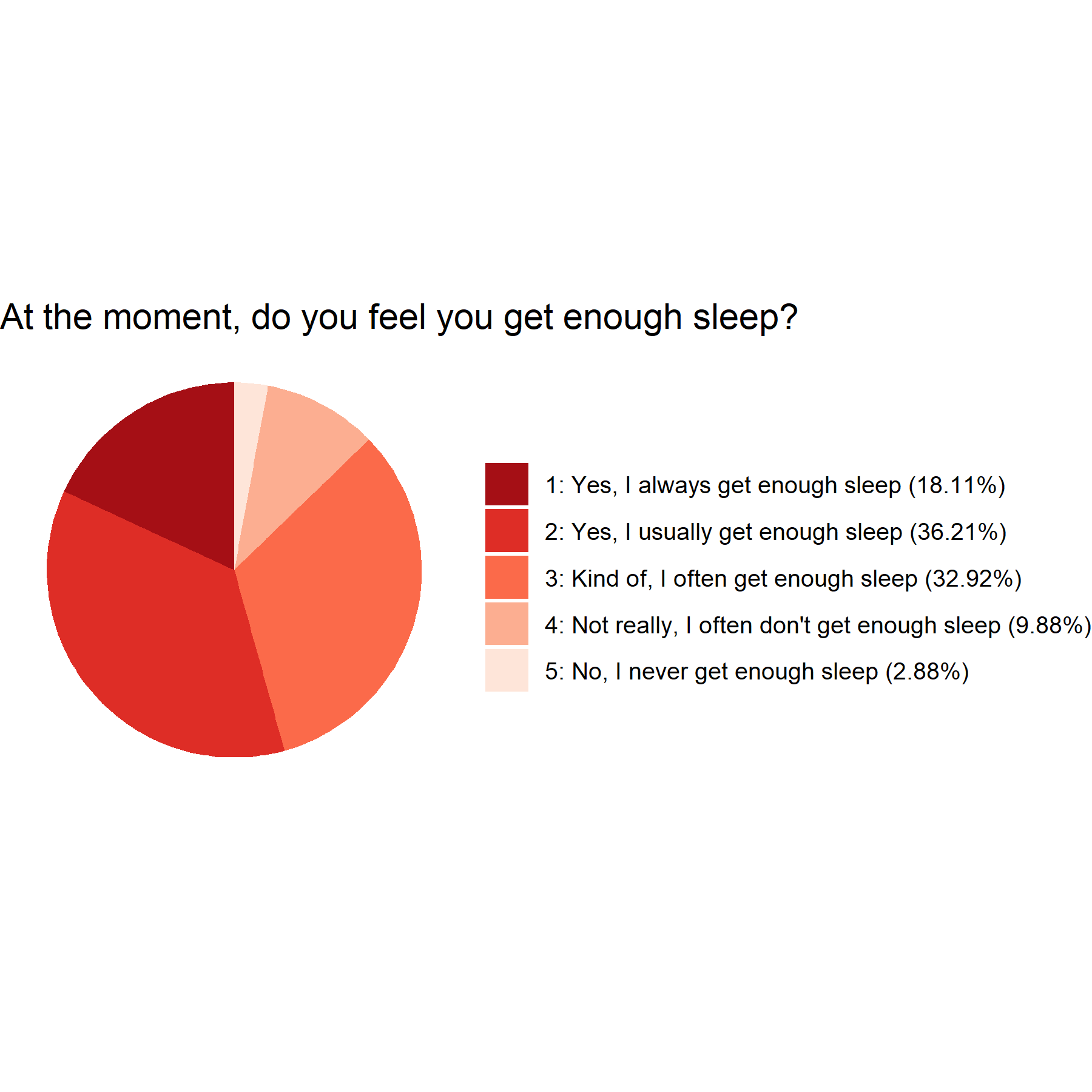


1. Phase 2


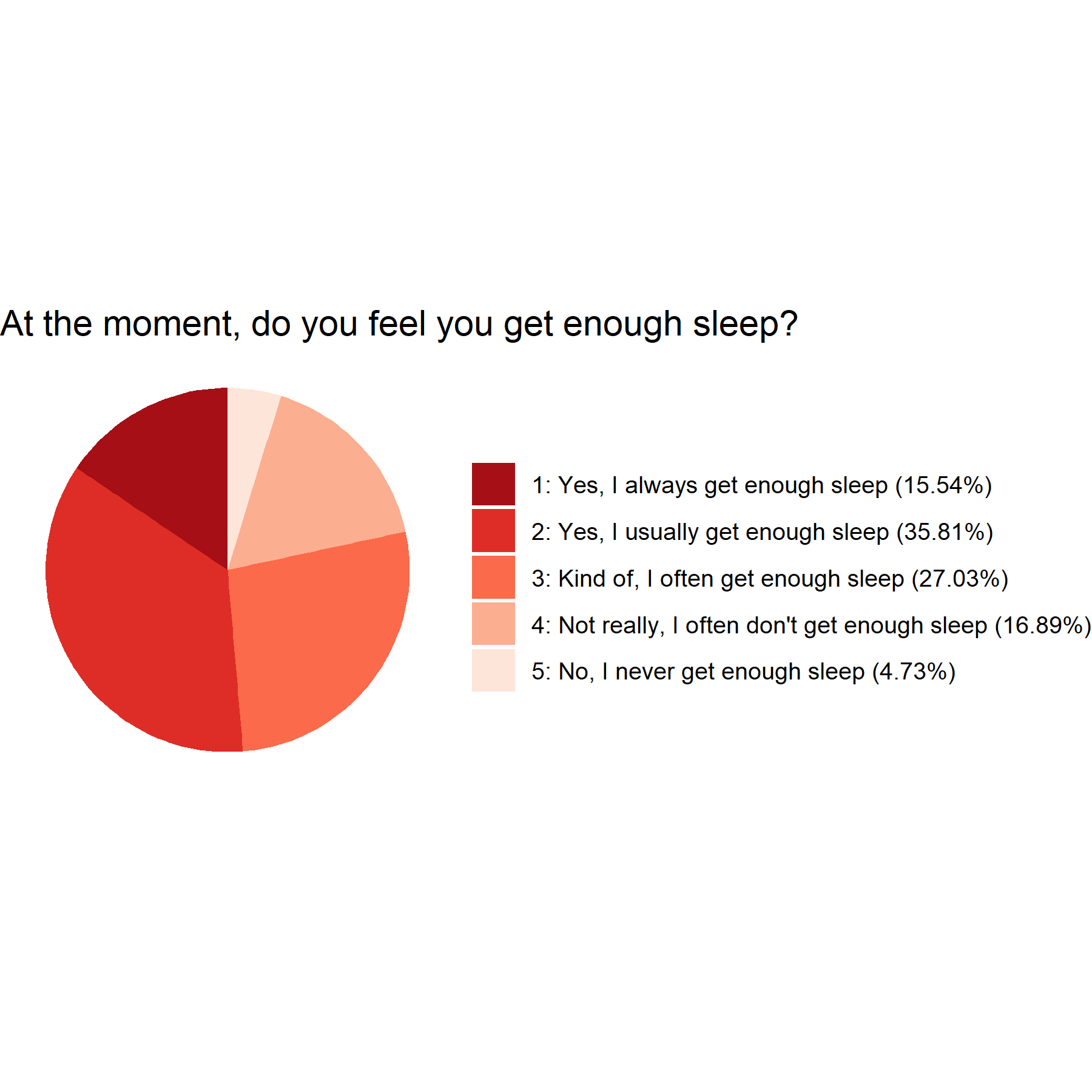

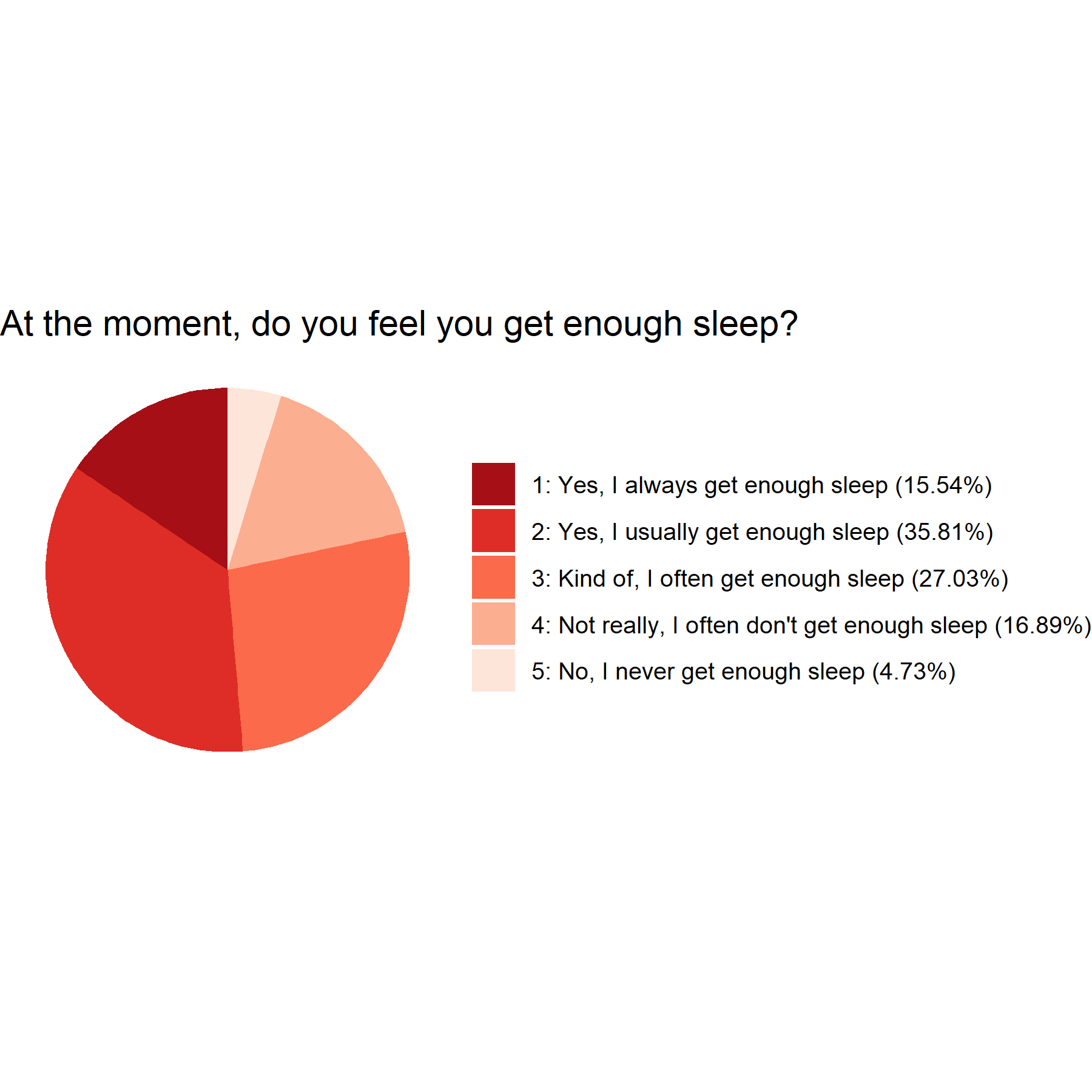


*After your lights are turned off, do you spend a long time thinking or worrying about things?*

1. Phase 2
2. Phase 1


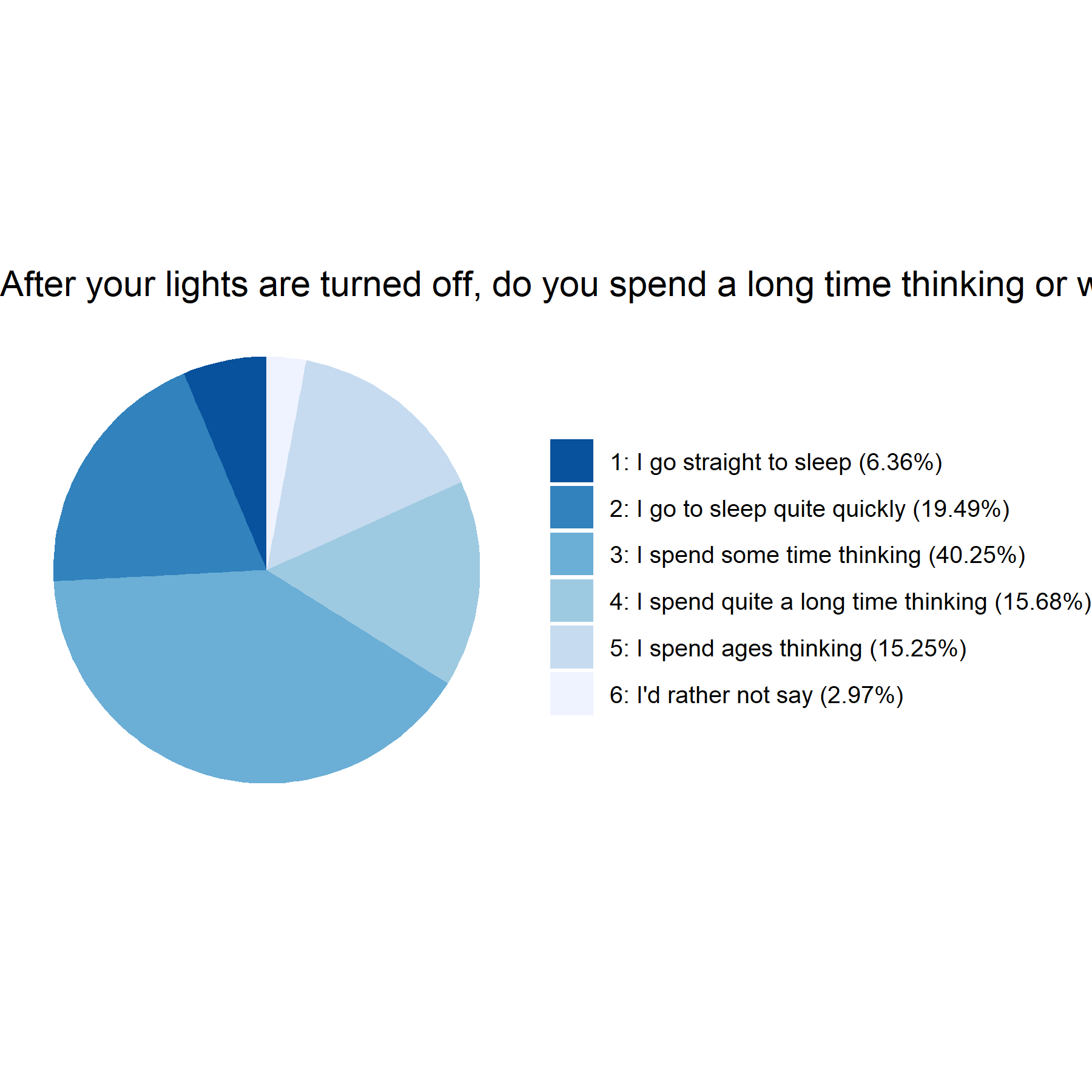

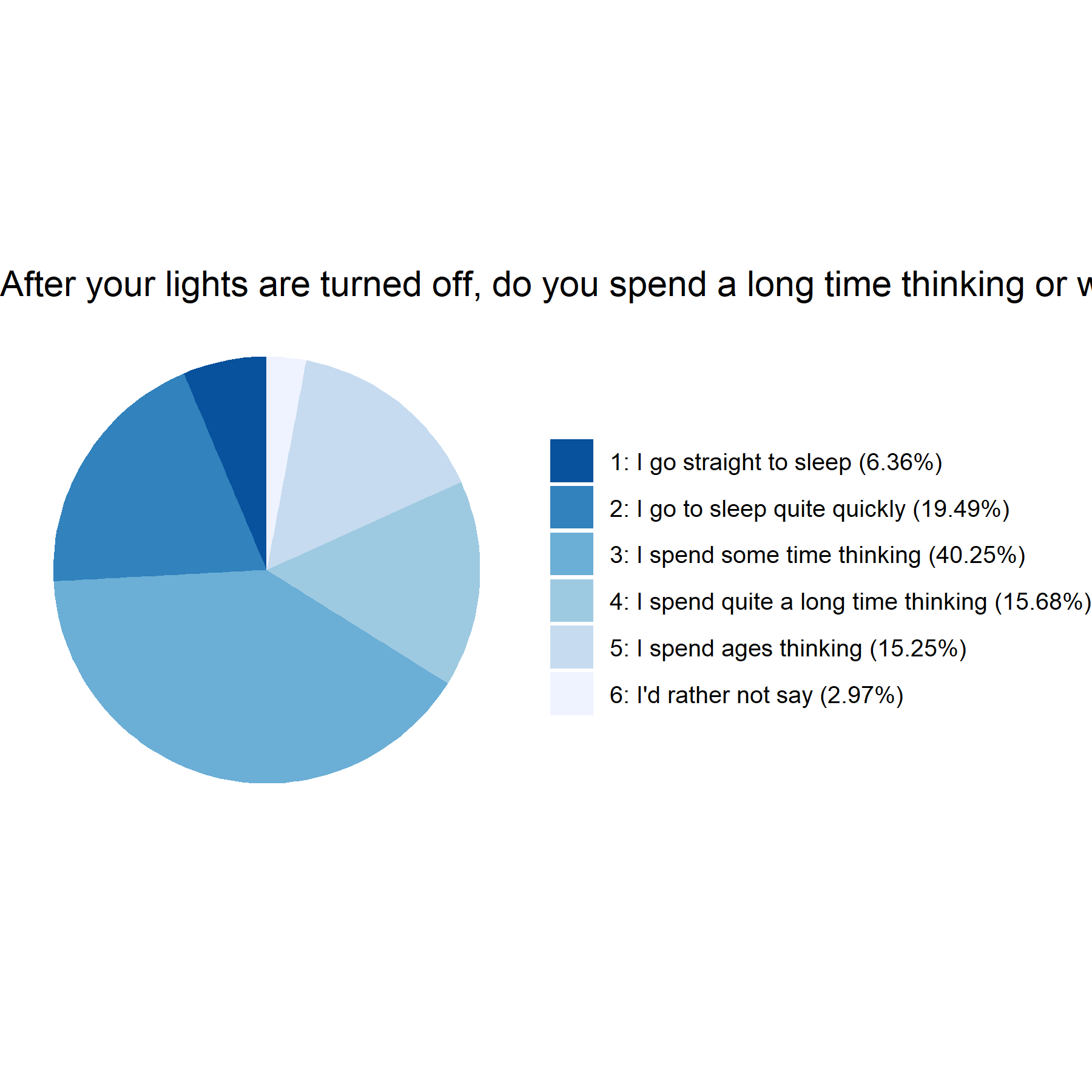


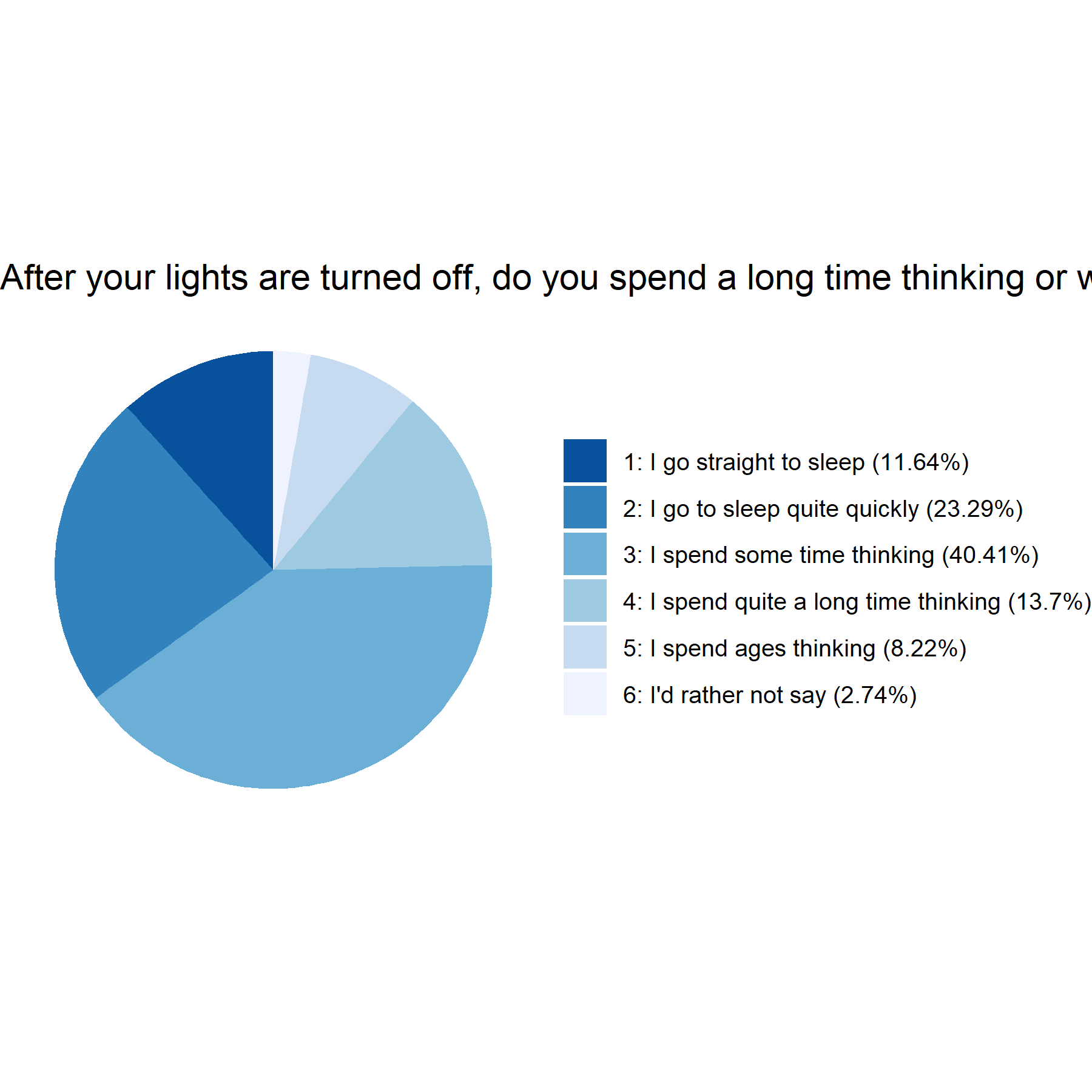

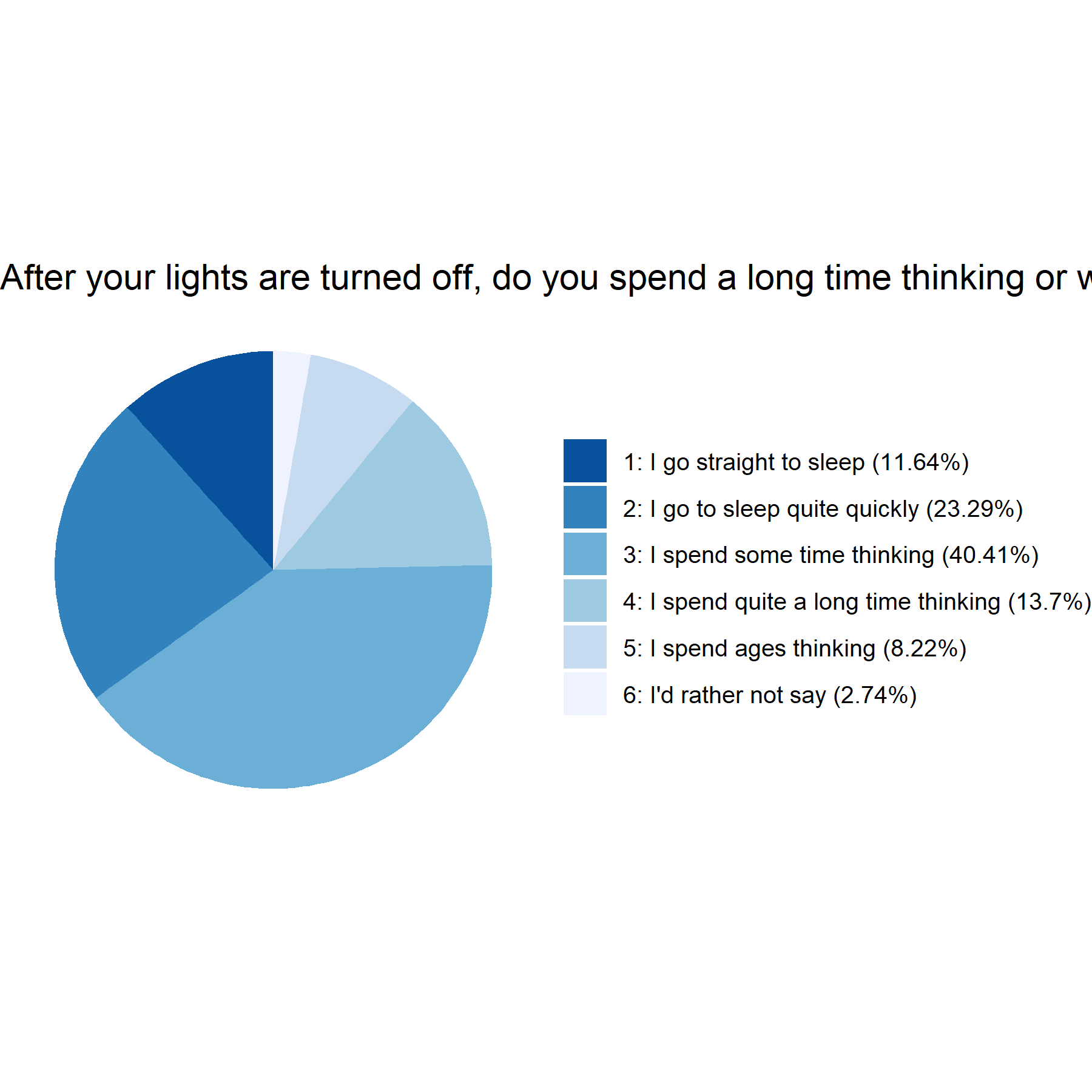


*If you wake in the night does it take you a long time to get back to sleep?*

1. Phase 1


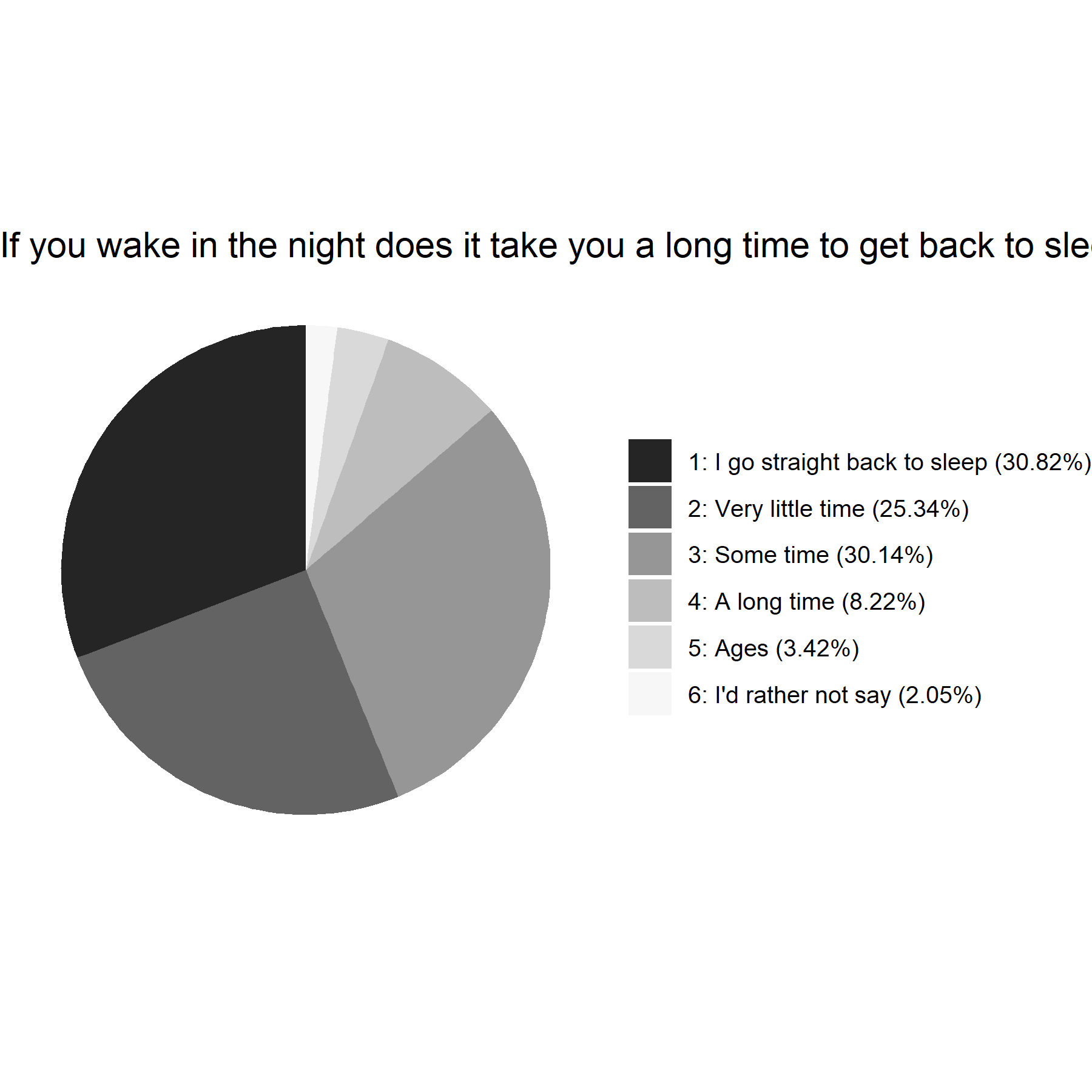


1. Phase 2


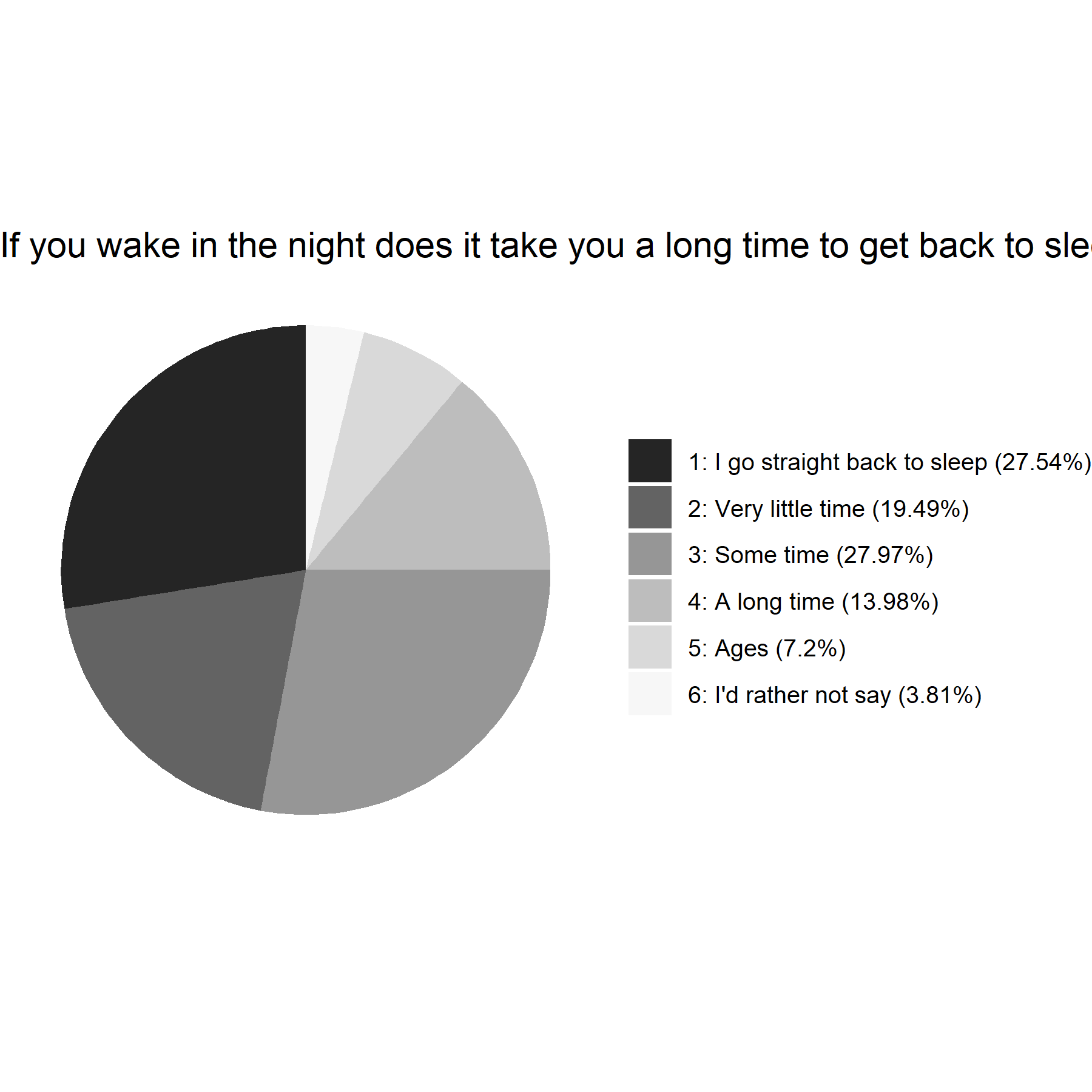

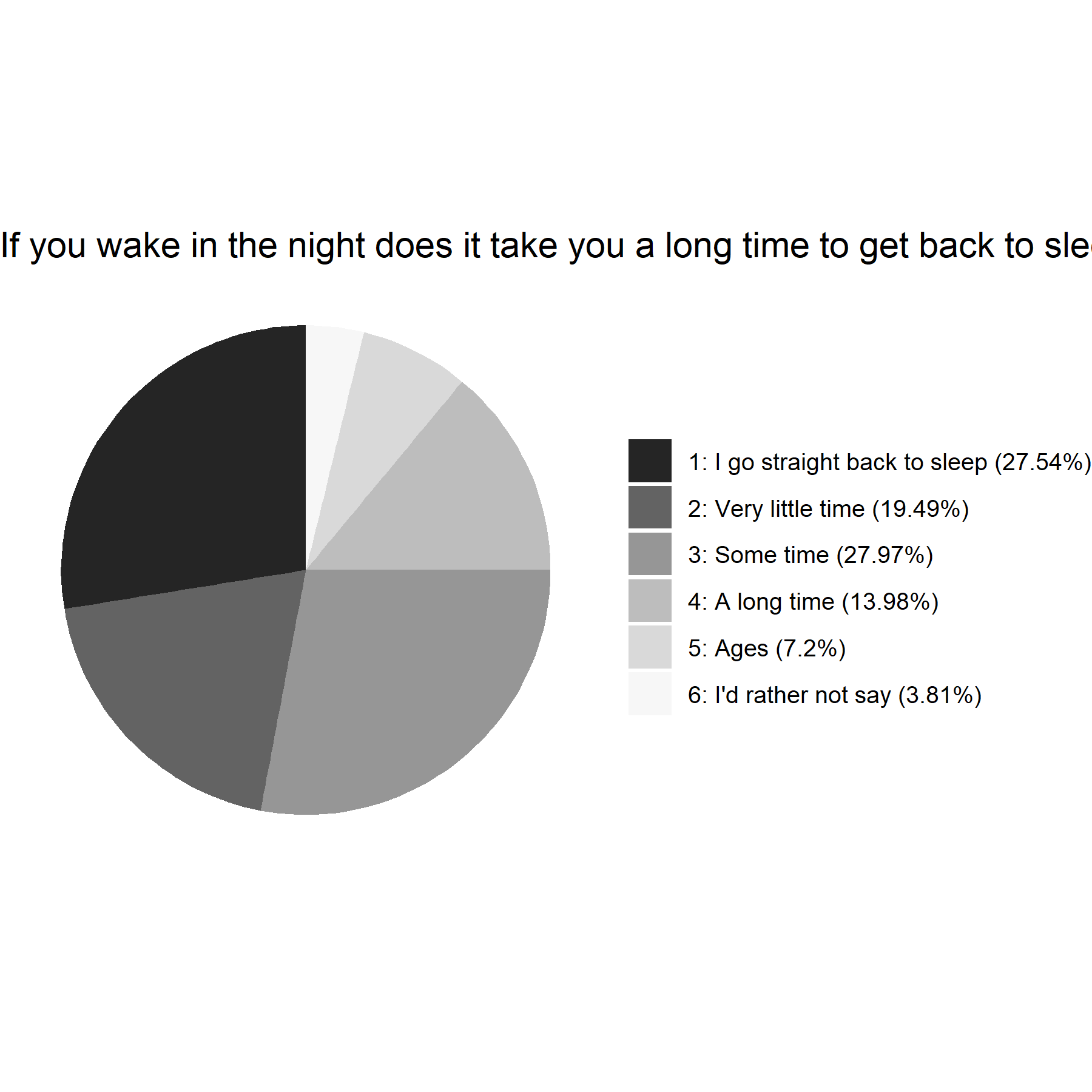

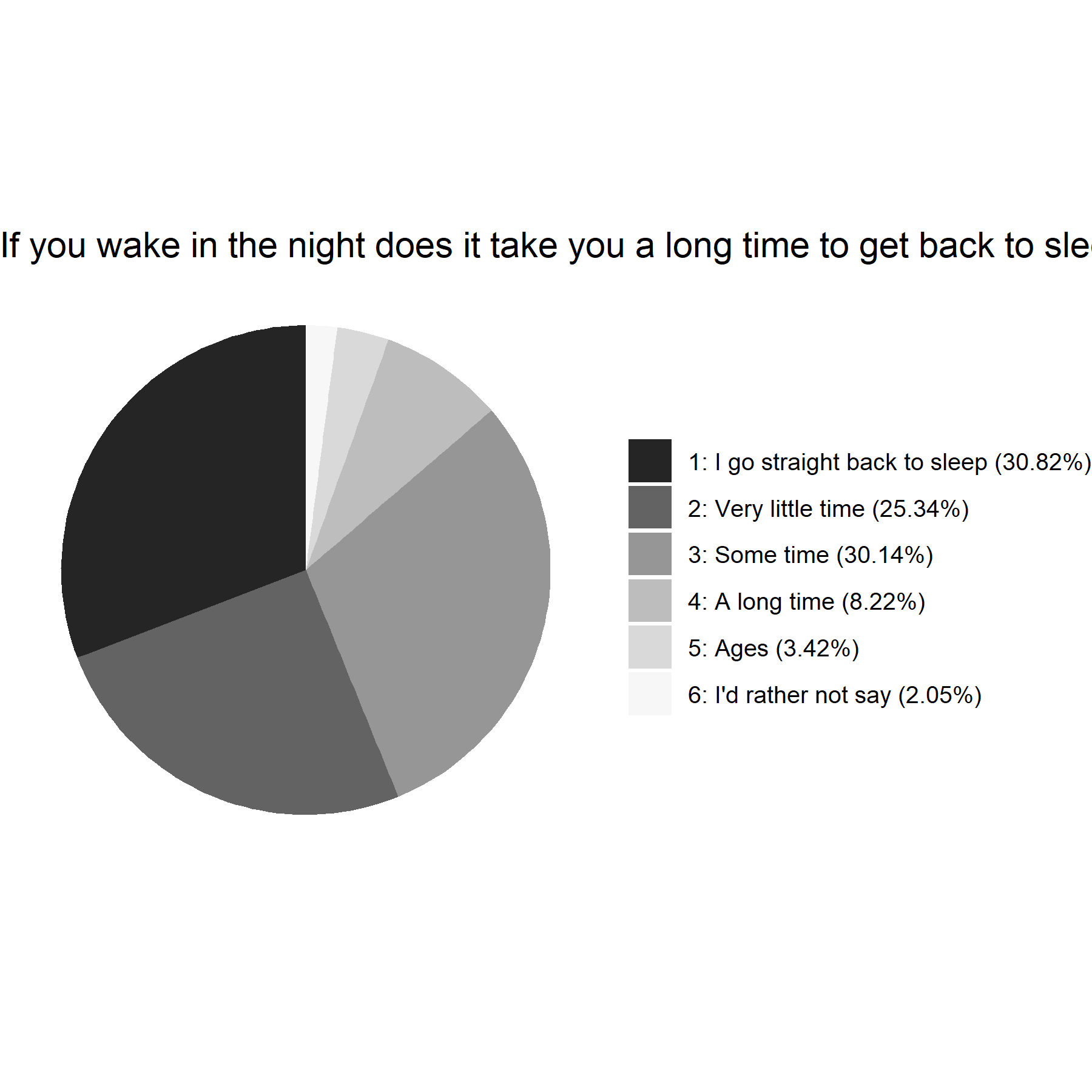


*At the moment, how sleepy do you feel during the day?*

1. Phase 1


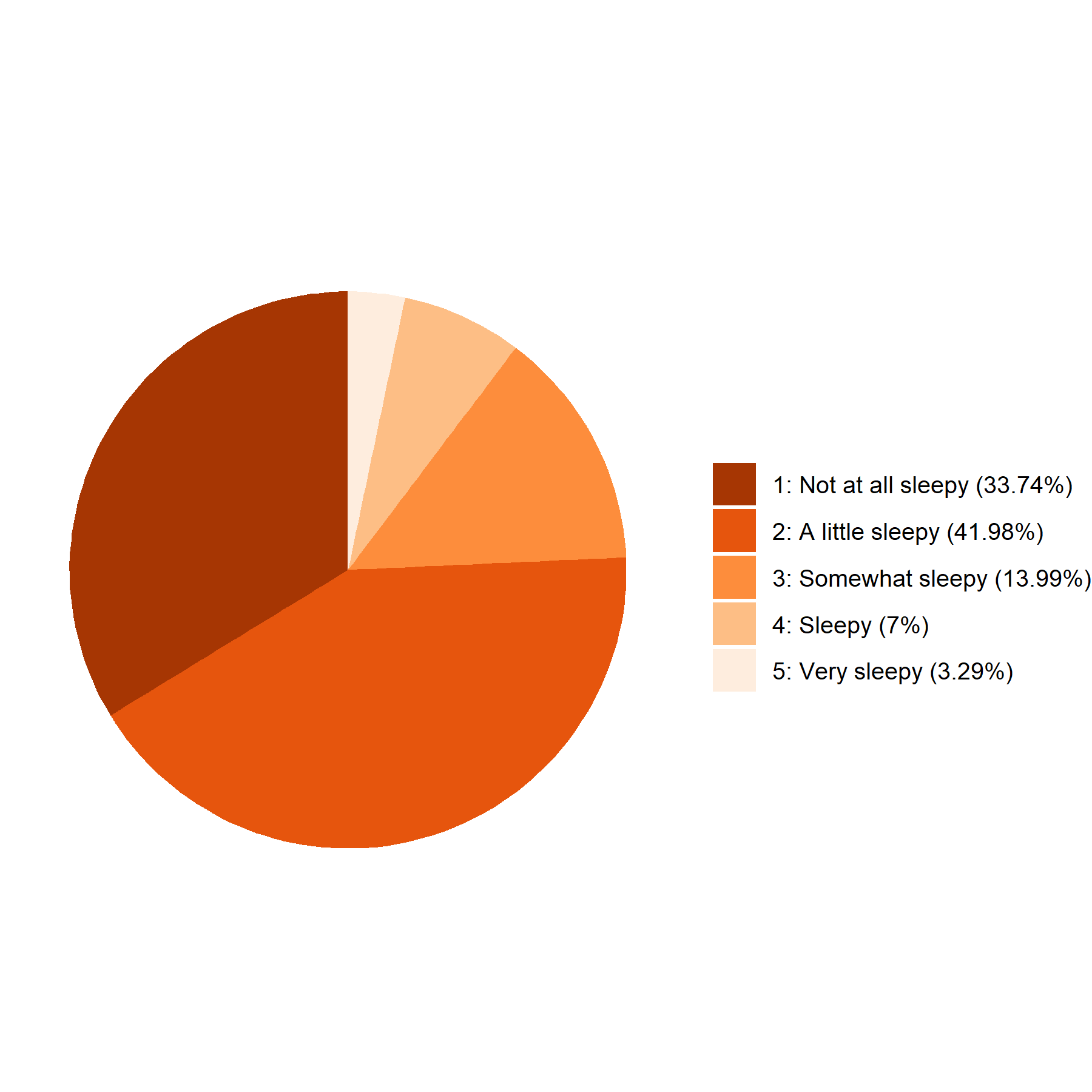


1. Phase 2


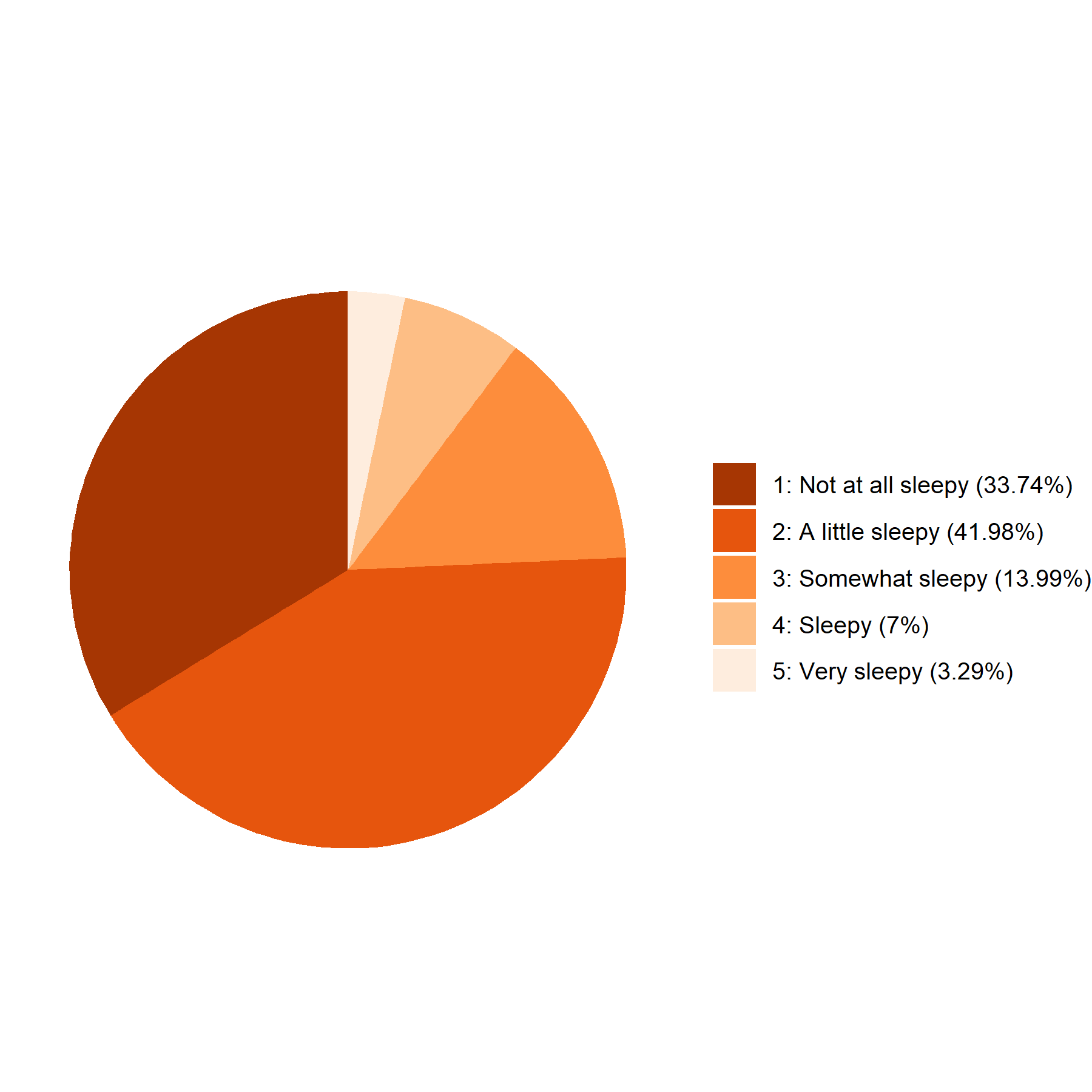

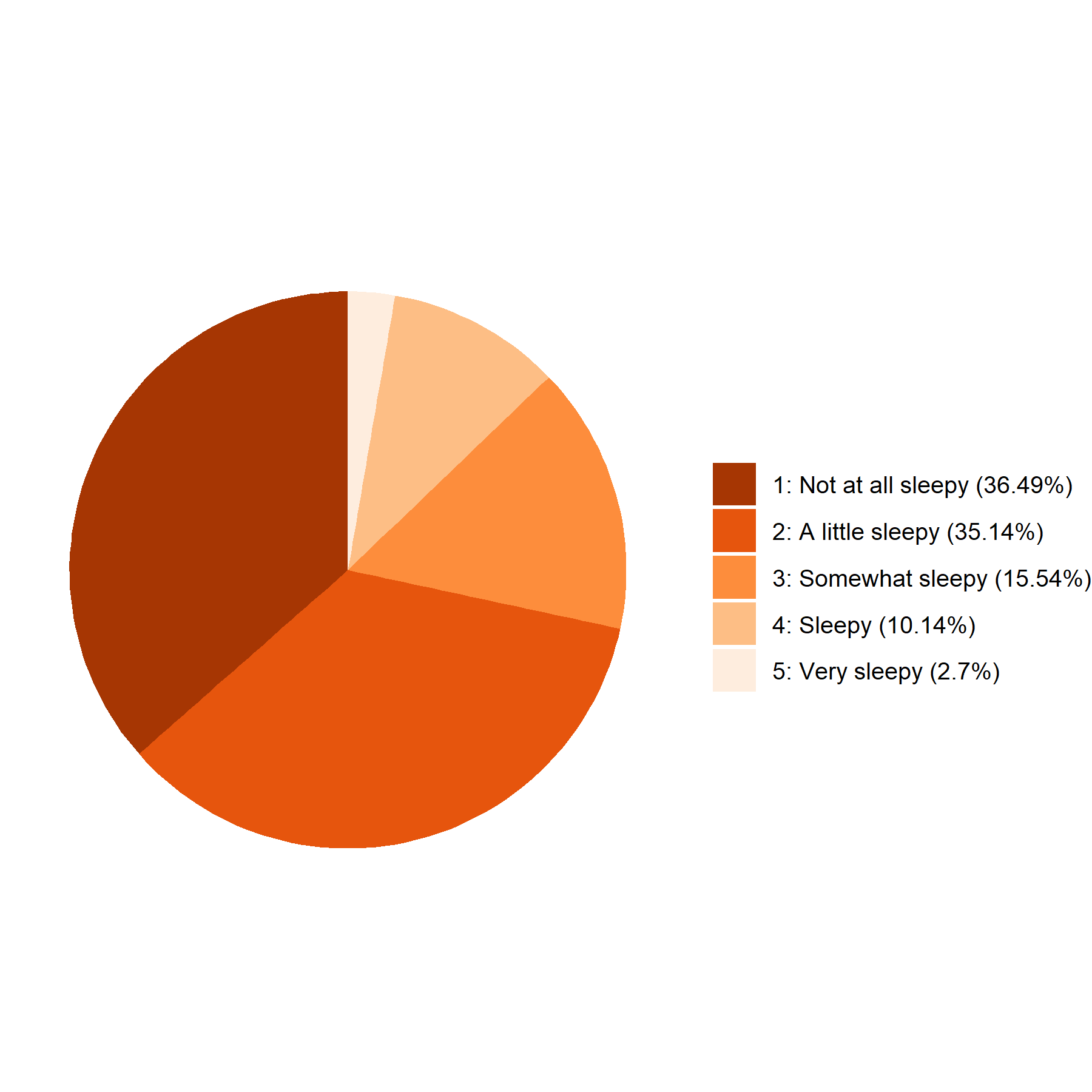

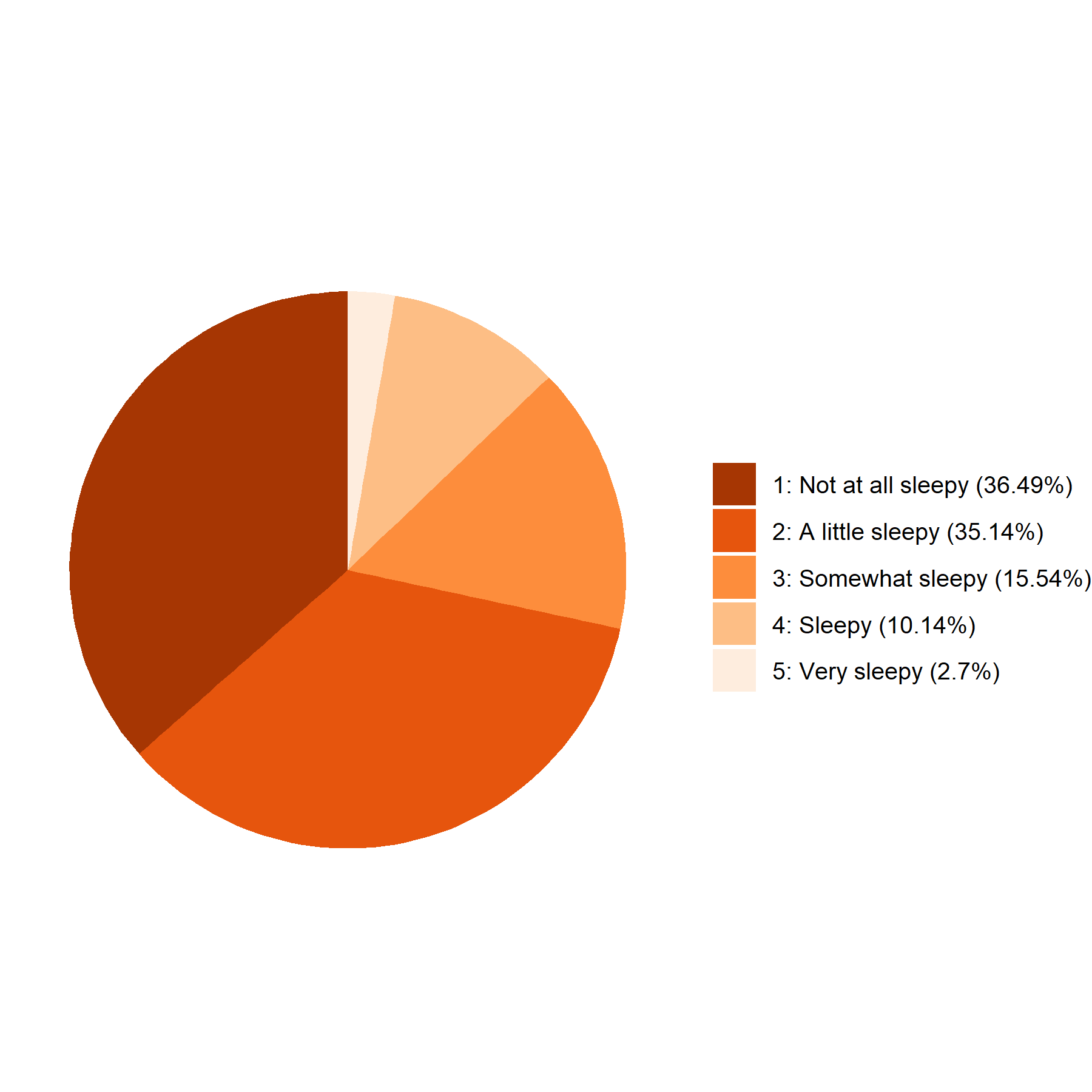


**Figure A1.** Responses to the questions posed to children at A/C/E/G) Phase 1 (n = 243) and B/D/F/H) Phase 2 (n = 148).

|  | *During the past week, did you feel scared or worried for no particular reason?* | | *During the past week, did you worry about the virus?* | |
| --- | --- | --- | --- | --- |
|  | **Phase 1**  **n = 145** | **Phase 2**  **n = 145** | **Phase 1**  **n = 134** | **Phase 2**  **n = 134** |
| ‘Never’ (%) | 37.0 | 39.8 | 28.8 | 42.8 |
| ‘Rarely’ (%) | 18.1 | 15.1 | 22.2 | 13.9 |
| ‘Sometimes’ (%) | 24.7 | 23.5 | 28.8 | 23.5 |
| ‘Quite often’ (%) | 10.3 | 6.0 | 8.6 | 4.8 |
| ‘Very often’ (%) | 2.8 | 1.8 | 5.3 | 1.8 |
| ‘I’d rather not say’ (%) | 3.7 | 1.2 | 2.9 | 0.6 |
| No response (%) | 3.3 | 1.3 | 3.3 | 1.3 |

**Table A2.** Responses to children’s questions about anxiety at Phase 1 & Phase 2.

|  |  | Method | r/r_s_ | Lower 95%CI | Upper 95%CI | df | p |
| --- | --- | --- | --- | --- | --- | --- | --- |
| Parent reported | Bedtime | Pearson | .74 | .66 | .80 | 177 | <0.001*** |
|  | Get up time | Pearson | .70 | .62 | .77 | 177 | <0.001*** |
|  | SOL | Spearman | .56 | .43 | .70 | 177 | <0.001*** |
|  | Daytime sleepiness | Spearman | .47 | .33 | .60 | 177 | <0.001*** |
|  | Time spent outside in week | Pearson | .44 | .31 | .55 | 177 | <0.001*** |
|  | Time spent outside at weekend | Pearson | .48 | .36 | .59 | 177 | <0.001*** |
|  | Screen hours for education | Pearson | .26 | .12 | .39 | 177 | 0.001** |
|  | Screen hours for entertainment | Pearson | .40 | .27 | .52 | 177 | <0.001*** |
|  | TV before bed | Spearman | .47 | .33 | .60 | 177 | <0.001*** |
|  | Games before bed | Spearman | .47 | .35 | .60 | 177 | <0.001*** |
|  | Social media before bed | Spearman | .30 | .12 | .49 | 177 | <0.001*** |
|  | Bedtime anxiety | Spearman | .50 | .38 | .63 | 177 | <0.001*** |
|  | Expressed anxiety about COVID-19 | Spearman | .43 | .30 | .56 | 177 | <0.001*** |
|  | Parental anxiety about COVID-19 | Spearman | .47 | .33 | .60 | 177 | <0.001*** |
|  |  |  |  |  |  |  |  |
| Self-reported | Enough sleep | Spearman | .39 | .24 | .55 | 135 | <0.001*** |
|  | Daytime sleepiness | Spearman | .39 | .22 | .55 | 135 | <0.001*** |
|  | SOL | Spearman | .42 | .27 | .56 | 132 | <0.001*** |
|  | Number of night wakings | Pearson | .62 | .51 | .72 | 132 | <0.001*** |
|  | Time to sleep after night waking | Spearman | .38 | .23 | .54 | 132 | <0.001** |
|  | General worry in surveyed week | Spearman | .36 | .21 | .51 | 132 | <0.001*** |
|  | COVID-19 worry in surveyed week | Spearman | .43 | .28 | .58 | 132 | <0.001*** |

**Table A3.** Correlations between comparable variables at Phase 1 and Phase 2.

**References**

**1.** Ford, T., Goodman, R., Meltzer, H. (2003). The British child and adolescent mental health survey 1999: prevalence of the DSM-VI disorders. *Journal of the American Academy of Child & Adolescent Psychiatry, 42* (10): 1203-1211. Doi: 10.1097/00004583-200310000-00011

**2.** Baron-Cohen, S., Scott, F.J., Williams, J., Bolton, P., Matthews, F. E., & Brayne, C. (2009). Prevalence of autism-spectrum conditions: UK school-based population study. *The British Journal of Psychiatry, 194*: 500–509. doi: 10.1192/bjp.bp.108.059345

**3.** Norbury, C.F., Gooch, D., Wray, C., Baird, G., Charman, T., Simonoff, E., Vamvakas, G., & Pickles, A. (2016). The impact of nonverbal ability on prevalence and clinical presentation of language disorder: evidence from a population study. *The Journal of Child Psychology & Psychiatry, 57* (11): 1247-1257. doi: 10.1111/jcpp.12573

**4.** Snowling, M. (2000). *Dyslexia* (2^nd^ Ed.). Blackwell, UK
